# Delimitation of the wild banana *Musa acuminata* subspecific complex (Musaceae) in Thailand

**DOI:** 10.3897/phytokeys.278.165557

**Published:** 2026-08-14

**Authors:** Wandee Inta, Sasivimon Chomchalow Swangpol, Kanokporn Athawongsa, Sirapope Wongniam, Narongsak Sukkaewmanee, Tosak Seelanan, Peerakitt Srikrainoon, Wipawee Nilapaka, Tiwa Rotchanapreeda, Jamorn Somana

**Affiliations:** 1 Department of Plant Science, Faculty of Science, Mahidol University, Bangkok 10400, Thailand Department of Plant Science, Faculty of Science, Mahidol University Bangkok Thailand; 2 Department of Medical Sciences, Ministry of Public Health, Nonthaburi 11000, Thailand The Medicinal Plant Information Center, Faculty of Pharmacy, Mahidol University Bangkok Thailand; 3 The Medicinal Plant Information Center, Faculty of Pharmacy, Mahidol University, Bangkok 10400, Thailand Research and Innovation Division, Faculty of Science, Mahidol University Bangkok Thailand; 4 Research and Innovation Division, Faculty of Science, Mahidol University, Bangkok 10400, Thailand Faculty of Tropical Medicine, Mahidol University Bangkok Thailand; 5 Art Education Section, Faculty of Anthropology and Social Science, Muban Chombueng Rajabhat University, Ratchaburi 70150, Thailand Department of Biochemistry, Faculty of Science, Mahidol University Bangkok Thailand; 6 Plants of Thailand Research Unit, Department of Botany, Faculty of Science, Chulalongkorn University, Bangkok 10330, Thailand Plants of Thailand Research Unit, Department of Botany, Faculty of Science, Chulalongkorn University Bangkok Thailand; 7 Faculty of Tropical Medicine, Mahidol University, Bangkok 10400, Thailand Art Education Section, Faculty of Anthropology and Social Science, Muban Chombueng Rajabhat University Ratchaburi Thailand; 8 Department of Biochemistry, Faculty of Science, Mahidol University, Bangkok 10400, Thailand Department of Medical Sciences, Ministry of Public Health Nonthaburi Thailand

**Keywords:** Biogeographic distribution, diagnostic characters, *Musa acuminata*, subspecific classification, taxonomy

## Abstract

Thailand, a recognized cradle of diversity for the wild banana *Musa acuminata*, has long been considered home to four subspecies: *siamea*, *burmannica*, *malaccensis*, and *microcarpa*. However, recent extensive field surveys across the country have unveiled a more complex picture, necessitating a revision of their taxonomic classification. This study revisits the subspecific classification of *M. acuminata* in Thailand, reassessing the circumscriptions, descriptions, and distributions of previously recognized subspecies using morphological and biogeographic evidence. The findings reveal a distinct new subspecies, *M. acuminata* subsp. *kraburiensis*, located on the Isthmus of Kra. This subspecies is distributed in the transitional zone between *M. acuminata* subsp. *siamea* in northern Thailand and *M. acuminata* subsp. *malaccensis* in Peninsular Thailand. Furthermore, a northern occurrence of the latter subspecies was recorded, suggesting a potential dispersal route along the Thailand–Myanmar mountain range. Fieldwork revealed additional novel forms within existing subspecies, i.e., a putative mutant form with yellow inflorescence bracts and another form with unusual branching of the inflorescence. Finally, *M. acuminata* subsp. truncata was recorded for the first time in Thailand. *Musa acuminata* subsp. *burmannica*, *M. acuminata* subsp. *burmannicoides*, and *M. acuminata* subsp. *microcarpa* were not observed during the field survey. Based on the analysis of the *M. acuminata* subspecies complex in Thailand, the variation is best treated at the subspecies rather than the varietal level. A key for identifying *M. acuminata* subspecies in Thailand is presented here based on diagnostic characters such as leaf base shape, rachis position, imbrication type, and color of the male bracts.

## Introduction

*Musa acuminata* Colla is native to tropical Asia. It constitutes one of the two progenitors of most seedless banana cultivars (Cavendish; AAA Group). Several subspecies have been described based on their morphology, geographic distribution, cytology, and molecular biology (Cheesman 1948; Simmonds 1956, 1962; Perrier et al. 2009; Rouard et al. 2018; Šimoníková et al. 2022; Martin et al. 2023). Based on morphology and geographic distribution, Perrier et al. (2009) suggested nine subspecies of *M. acuminata*: subsp. *banksii*, subsp. *burmannica*, subsp. *burmannicoides*, subsp. *errans*, subsp. *malaccensis*, subsp. *microcarpa*, subsp. *siamea*, subsp. truncata, and subsp. *zebrina*, as well as three varieties: var. *chinensis*, var. *sumatrana*, and var. *tomentosa*.

Due to the large size and herbaceous habit of bananas, specimens are often deficient and difficult to compare (Nasution 1991; Wong et al. 2001). Several attempts have been made to describe the variation in *M. acuminata* at the subspecific level. Thus, Simmonds (1956) classified the complex using a combination of vegetative, floral, fruit, and ovary characters. The key primarily relied on sheath waxiness, pseudostem pigmentation, and several male bract features, including color, imbrication, persistence, and the presence of yellowish tips. Additional diagnostic characters included the fertility of female flowers, fruit shape (subobtuse vs. acuminate), and the number of ovules per ovary. Among these, male-phase inflorescence characters—particularly bract morphology and coloration—played a central role in delimiting subspecies. Later, De Langhe and Devreux (1960) used bract color, the pedicel, apex, and cross section of the fruit, bunch direction, and floral and fruit density as diagnostic features and erected a new subspecies, *Musa acuminata* subsp. *burmannicoides* De Langhe & Devreux, distributed in India. Nasution (1991) used reproductive, inflorescence, vegetative, and biogeographic characters to discriminate among 15 varieties of *M. acuminata* in Indonesia, including *acuminata*, *microcarpa* (Becc.) Nasution, and *malaccensis* (Ridl.) Nasution. The key relied primarily on floral and seed traits, including the sex of basal flowers, seed morphology, fruit indumentum, tepal shape, and staminode length, supplemented by male-phase inflorescence and bract morphology, leaf pigmentation, and geographic distribution. Later, De Langhe et al. (2000) reported an unknown form, informally referred to as pseudo-malaccensis, as well as several other allegedly subspecific hybrids within the *M. acuminata* populations found in northern Thailand. The identities of *M. acuminata* subsp. *malaccensis* (Ridl.) N.W.Simmonds, *M. acuminata* subsp. *microcarpa* (Becc.) Simmonds, and *M. acuminata* subsp. truncata (Ridl.) Kiew were also considered dubious (Häkkinen and De Langhe 2001) until Wong et al. (2001) clarified that they were genetically distinct taxa. Wong et al. (2001) emphasized the significant spatial separation of the subspecies. Thus, subsp. *malaccensis* and subsp. *microcarpa* occur in the lowlands at 600–900 m, whereas subsp. truncata is usually found above 900 m in mountainous areas. The subspecies *malaccensis* and truncata are distributed in mainland Malaysia, whereas subsp. *microcarpa* is restricted to the island of Borneo. Subsequent molecular analyses confirmed the status of these three subspecies (Wong et al. 2001).

Thailand is located in the transitional zone between the Indochinese and Sundaic biogeographic regions (Tougard 2001). Cheesman (1948: 27) noted that “the range of variation of the (*M. acuminata*) species in Siam (Thailand) is of great importance and much needs further study.” According to Simmonds (1960), the three *M. acuminata* subspecies—subsp. *siamea* Simmonds, subsp. *burmannica* Simmonds, and subsp. *malaccensis* (Ridl.) Simmonds—are widely distributed from southern India to Indochina in the north and from southern China to Borneo in the east. They overlap in Thailand along the Thailand–Myanmar border, in southern Thailand, and in the Isthmus of Kra Archipelago. However, no thorough study of *M. acuminata* across the entire Kingdom of Thailand has been conducted since Simmonds’ surveys in the 1950s and those of De Langhe et al. (2000), both of which were biased toward the northern region. Subspecies *burmannica*, *burmannicoides*, and *siamea* constitute both a morphological and a genetic complex with a geographic distribution across northeastern India, Burma, southern China, and Thailand. Molecular and cytogenetic data indicate that these three subspecies should be merged into subsp. *burmannica* (Perrier et al. 2009; Martin et al. 2020, 2023). Based on genetic evidence, it seems that this complex is closely related to subsp. *malaccensis* (Perrier et al. 2011). Genome assemblies indicated hybridization between subsp. *burmannica* and subsp. *malaccensis*, which is supported by their distributional overlap (Perrier et al. 2011; Rouard et al. 2018).

In a race against time to collect and protect valuable germplasm, several hundred *M. acuminata* accessions were collected and investigated throughout Thailand. This work has increased insight into the taxonomy of *Musa acuminata* at the subspecific level. A novel identification key to the subspecies of the *M. acuminata* complex in Thailand is proposed here.

## Materials and methods

### Taxon sampling

**Figure 1.**
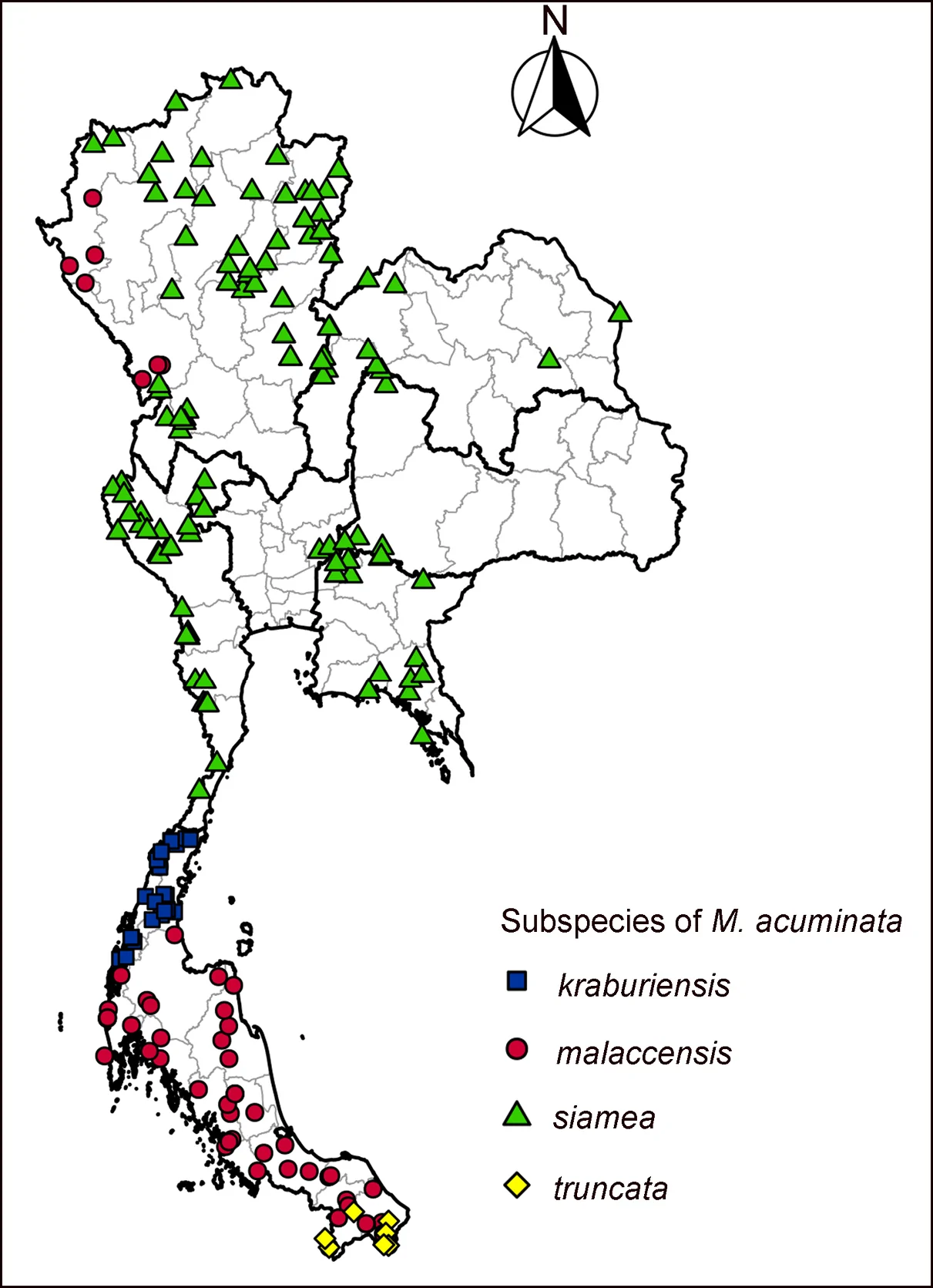
Distribution of the four subspecies of *Musa acuminata* across the floristic provinces of Thailand (Northern, North-eastern, Eastern, South-western, Central, South-eastern, and Peninsular). Symbols represent records of each subspecies.

All specimens were collected during several field campaigns in Thailand from 2005 through 2010. Identifications were based on the keys and descriptions in Cheesman (1948), Simmonds (1956, 1962), De Langhe and Devreux (1960), and De Langhe et al. (2000). A total of 211 accessions of *Musa acuminata* Colla *sensu lato* were studied, all of which were collected in natural habitats (Suppl. material 1). The outgroup comprised three accessions of wild *M. itinerans* Cheesman and six accessions of wild *M. rubra*. All reference collections were deposited at the SLR Herbarium in Bangkok (Holmgren et al. 1990). The voucher specimens included the apical part of the inflorescence (male flowers) and fruits preserved in alcohol, as well as the base, middle, and apex of an untorn mature leaf. Morphological descriptions were prepared on-site using a standardized format that integrated the diagnostic features suggested by Simmonds and Shepherd (1955), De Langhe and Devreux (1960), Simmonds (1962), Argent (1976), and Nasution (1991) and are available from the authors. Colors were determined using the IPGRI-INIBAP/CIRAD color chart for bananas (IPGRI-INIBAP/CIRAD 1996). All accessions were geolocated with a handheld GPS tracker. The circumscriptions of floristic regions and provinces (Changwats) follow those of the Flora of Thailand project (1970–2026) (Suppl. material 1; Fig. 1).

### Morphological analysis

A total of 16 morphological characters were scored in the field for 195 accessions. The characters were divided into states, as shown in Table 1, Suppl. material 2. Similarity coefficients were then calculated using PAST 4.05 (Hammer et al. 2001). All characters used in the analyses were equally weighted. The cluster analysis combined Gower’s distance measure with the unweighted pair-group method using arithmetic averages (UPGMA). Diagnostically useful characters for distinguishing subspecies of *M. acuminata* were derived by maximum parsimony mapping of character states onto the resulting dendrogram.

**Table 1.** Coding of 16 morphological characters for use in UPGMA multivariate analysis.

**#**	**Characters**	**State 1**	**State 2**	**State 3**	**State 4**	**State 5**
1	Petiolar canal	Open with margin spreading	Wide with erect margins	Straight with erect margins	Margins curved inward	Margins overlapping
2	Leaf base	Cuneate	Oblique	Round	Cordate	Auriculate
3	Peduncle and rachis color	Green	Purplish brown to dark purple			
4	Bunch direction	Horizontal to angle	Erect			
5	Number of rows in fruit hand	Uniseriate	Biseriate			
6	Rachis diameter	Slender (Diameter less than 2.5 cm)	Thick (diameter more than 2.5 cm)			
7	Rachis direction	Hanging vertically	At an angle	With a curve	Horizontal	Erect
8	Direction of inflorescence apex in the male phase	Vertically	Downward	Upward	Erect	
9	Male-phase inflorescence number	One	Numerous			
10	Bud shape	Ovate	Elliptic-ovate			
11	Arrangement of the rachis bracts	Greatly overlap	Slightly overlap	Non-overlap		
12	Inside (adaxial) color of the rachis bracts	Pink purple, red purple, orange red	Yellow	Ivory	Orange	
13	Outside (abaxial) color of the rachis bracts	Orange red, red, purple red, red purple, dark purple, purple brown	Orange	Yellow		
14	Shoulders of free tepal	Entire	Incised			
15	Fruit shape	Tubular	Fusiform			
16	Seed surface	Smooth	Rough			

## Results

**Figure 2.**
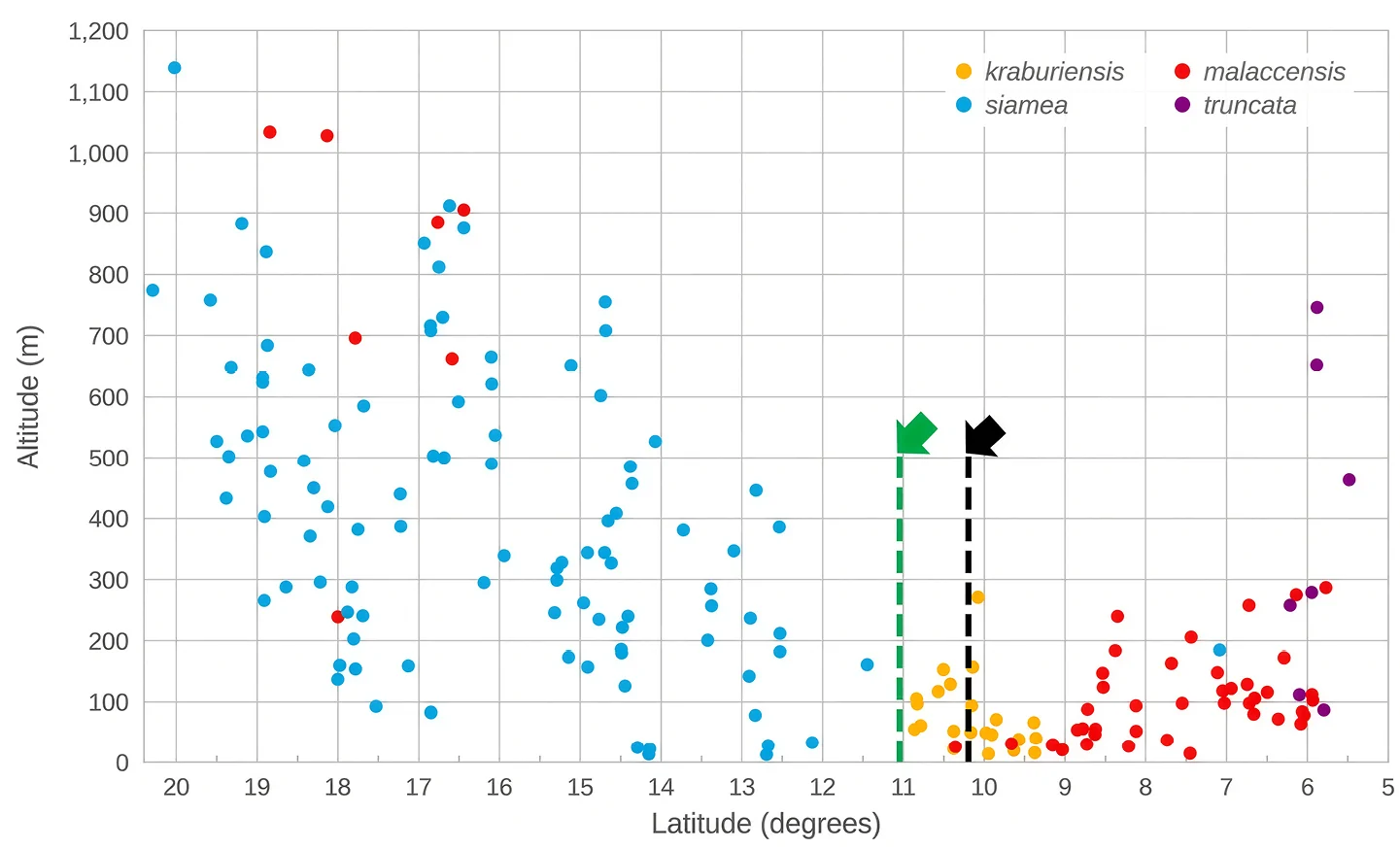
A two-dimensional plot of the 199 *Musa acuminata* accessions collected in Thailand as part of this study. The *x*-axis represents latitude, and the *y*-axis represents altitude. The four subspecies of *M. acuminata* are color-coded. The green dashed line and arrow indicate the transition between subsp. *siamea* and subsp. *kraburiensis* near the Isthmus of Kra at 10°11'N, latitude (black dashed line and arrow).

The evidence obtained from 211 accessions collected over 20 years revealed a remarkable diversity of *M. acuminata* throughout Thailand and allowed the geographic and altitudinal distributions of its subspecies to be mapped (Figs 1, 2). Only a few wild *Musa* species were recorded in northeastern and eastern Thailand, most probably because of forest clearing and human disturbance.

*Musa acuminata* subsp. *siamea* is the most widely distributed subspecies. It has predominantly been recorded on the central plain, with extensions northward, southeastward, and southwestward. *Musa acuminata* subsp. *kraburiensis*, a newly described subspecies recorded from sea level to 300 m altitude, has a restricted range between 9°N and 11°N. This corresponds to the transition between the Tanao Si/Tenasserim and Phuket mountain ranges on the Isthmus of Kra in the provinces of Chumphon and Ranong. Subspecies *malaccensis* is distributed throughout Peninsular Thailand. It was also recorded in northern Thailand at elevations of up to 1,000 m, which is considerably higher than the previously reported 300 m. The fourth subspecies, *M. acuminata* subsp. truncata, is newly recorded for Thailand. It was observed along the Thai border with Malaysia in the Sankalakhiri and Titiwangsa mountain ranges in the provinces of Narathiwat and Yala. In this area, it occurs sympatrically with subsp. *malaccensis* at lower elevations, and several putative hybrids between these two subspecies were observed.

### Discriminating morphological characters

**Figure 3.**
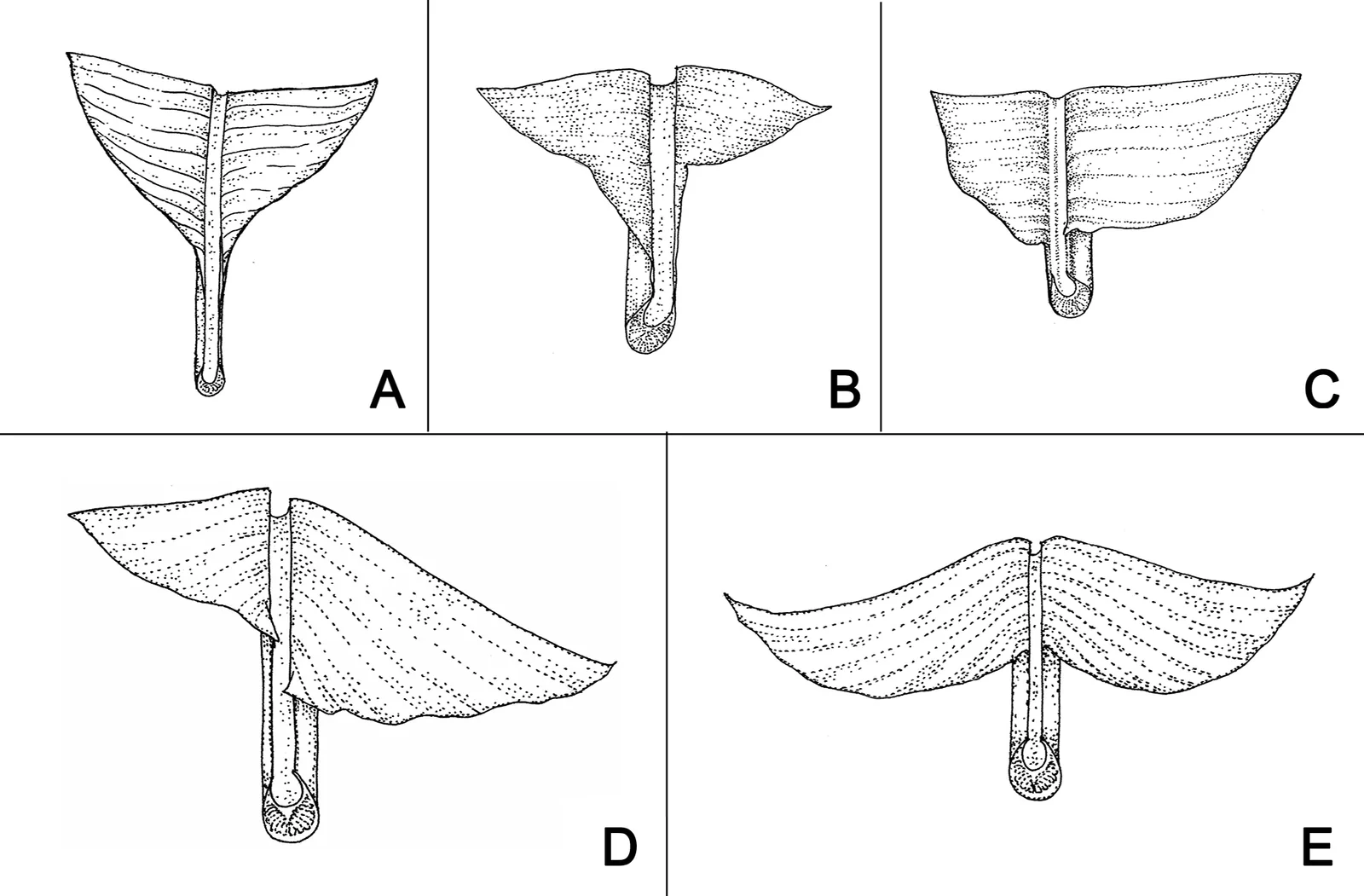
Variation in the leaf bases observed in the four subspecies of *Musa acuminata* recorded in Thailand. **A**. Cuneate—subsp. *siamea*, Swangpol & Somana 091, 182, 262; **B**. Oblique—subsp. *siamea*, Swangpol & Somana 021, 247, 266; **C**. Round—subsp. *kraburiensis*, Swangpol & Somana 107, 108, 142; subsp. *malaccensis*, Swangpol & Somana 204, 274; subsp. truncata, Swangpol & Somana 206, 490, 497; **D**. Auriculate—subsp. *kraburiensis*, Swangpol & Somana 009; subsp. *malaccensis*, Swangpol & Somana 005, 006, 010; **E**. Cordate—subsp. *malaccensis*, Swangpol & Somana 082, 138, 546. Illustrations by Potjana Kaittiprapai.

**Figure 4.**
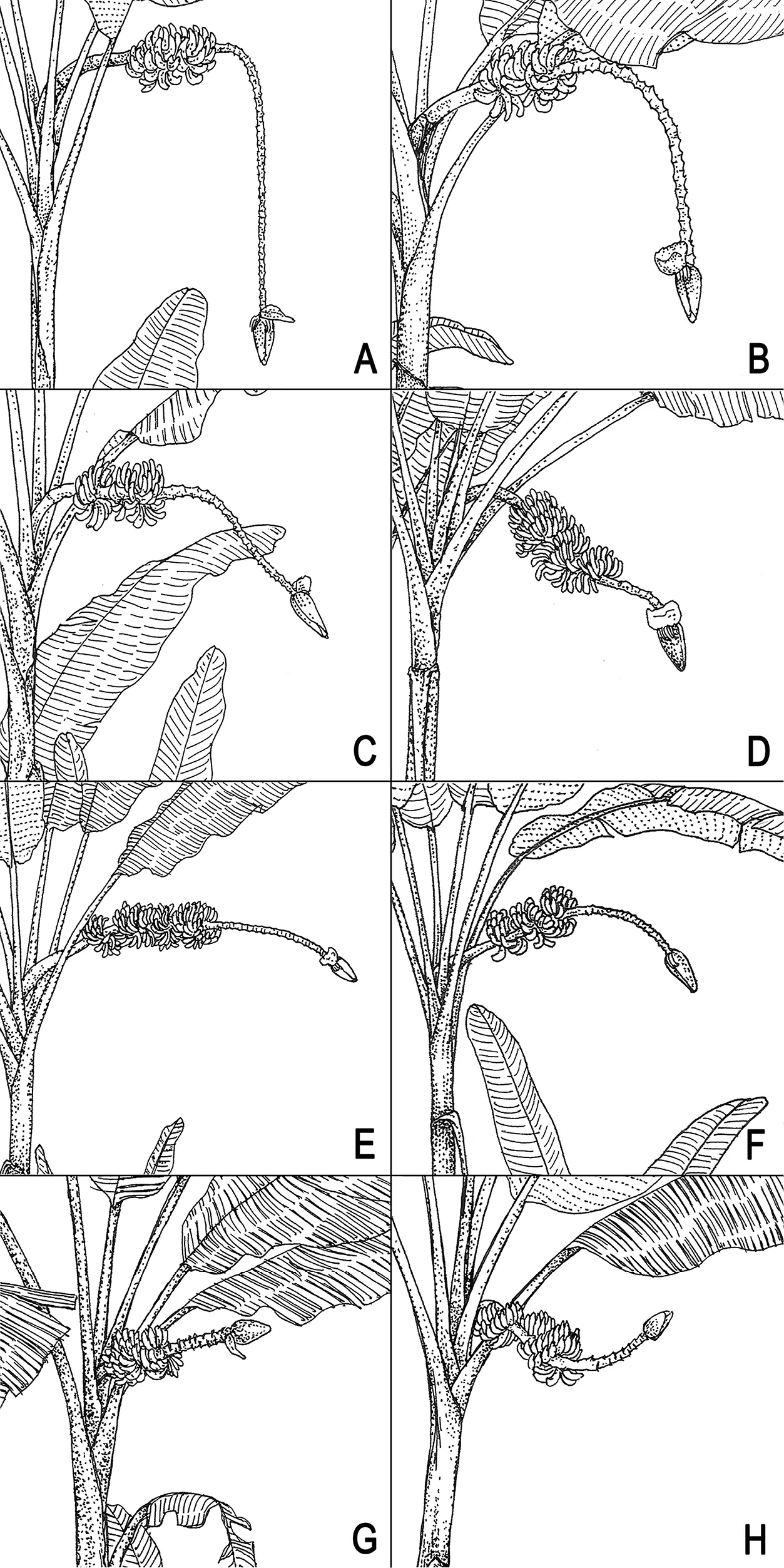
Variation in the position of the inflorescence/infructescence rachis observed in the four subspecies of *Musa acuminata* recorded in Thailand. **A, B**. Drooping at an angle—subsp. *siamea*, Swangpol & Somana 172, 007; **C**. Curved downward—subsp. *siamea*, Swangpol & Somana 247; **D**. Downward at an angle—subsp. truncata, Swangpol & Somana 206; **E, F**. Horizontal with slight (**E**) or pronounced curvature (**F**)—subsp. *malaccensis* (typical form), Swangpol & Somana 204, 203; **(G)** horizontal to pointing upward—subsp. *kraburiensis*, Swangpol & Somana 104; **H**. Curved upward—subsp. *kraburiensis*, Swangpol & Somana 107. Illustrations by Potjana Kaittiprapai.

**Figure 5.**
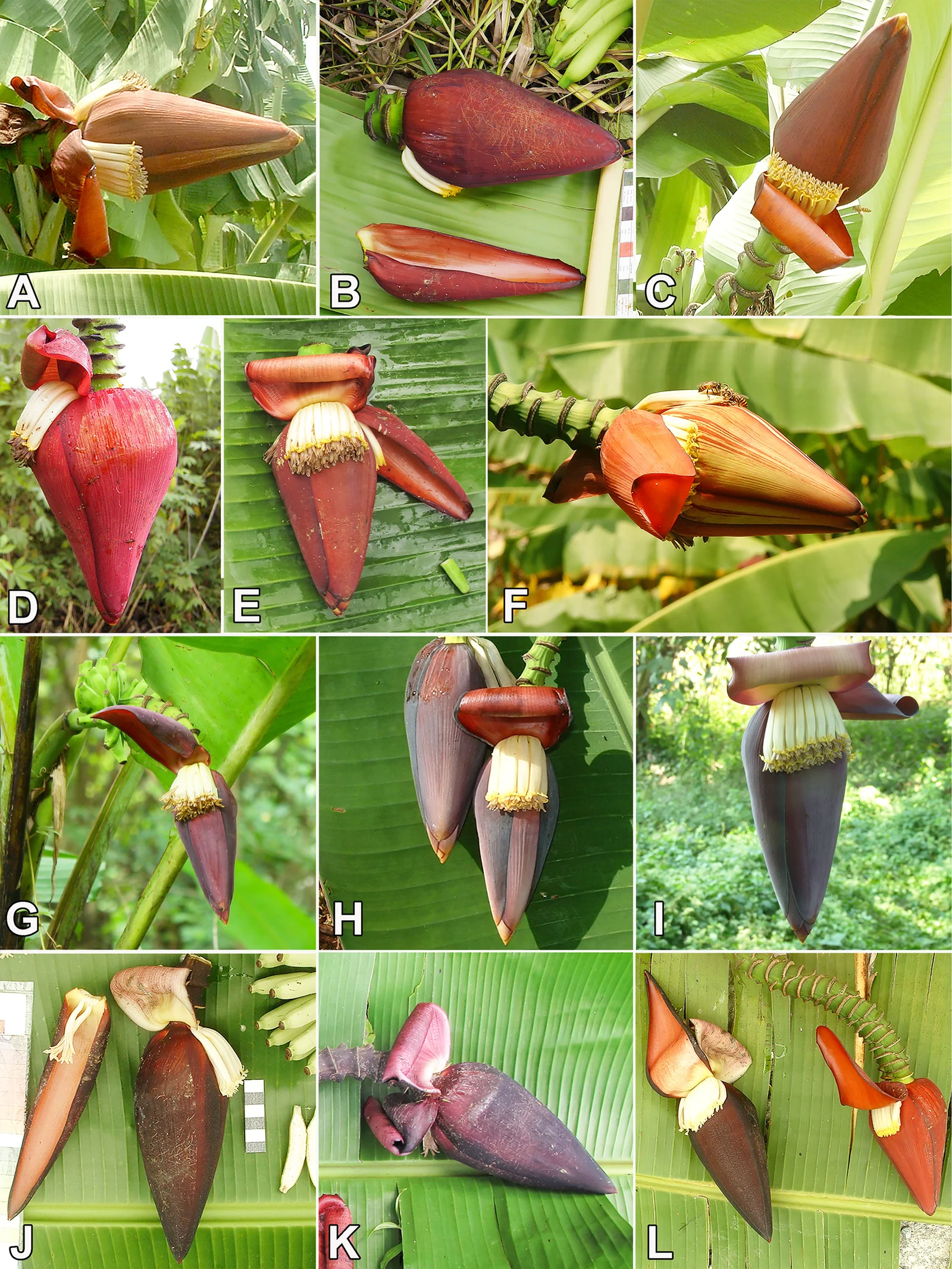
Variation in the overall shape, arrangement, and color of the apical male flower cluster observed across the four *Musa acuminata* subspecies recorded in Thailand: **A–C**. Subsp. *kraburiensis*—Swangpol & Somana 108, 107, 142; **D–F**. Subsp. *malaccensis*—Swangpol & Somana 140, 485, 138; **G–I**. Subsp. *siamea*—Swangpol & Somana 021, 091, 266; **J, K**. Subsp. truncata—Swangpol & Somana 491, 497; **L**. Comparison of subsp. truncata—Swangpol & Somana 206—and nearby subsp. *malaccensis* (no collection). The photos are not to scale, but the orientation of the apical male flower clusters corresponds to their position in the inflorescence. Photos by Sasivimon C. Swangpol.

**Figure 6.**
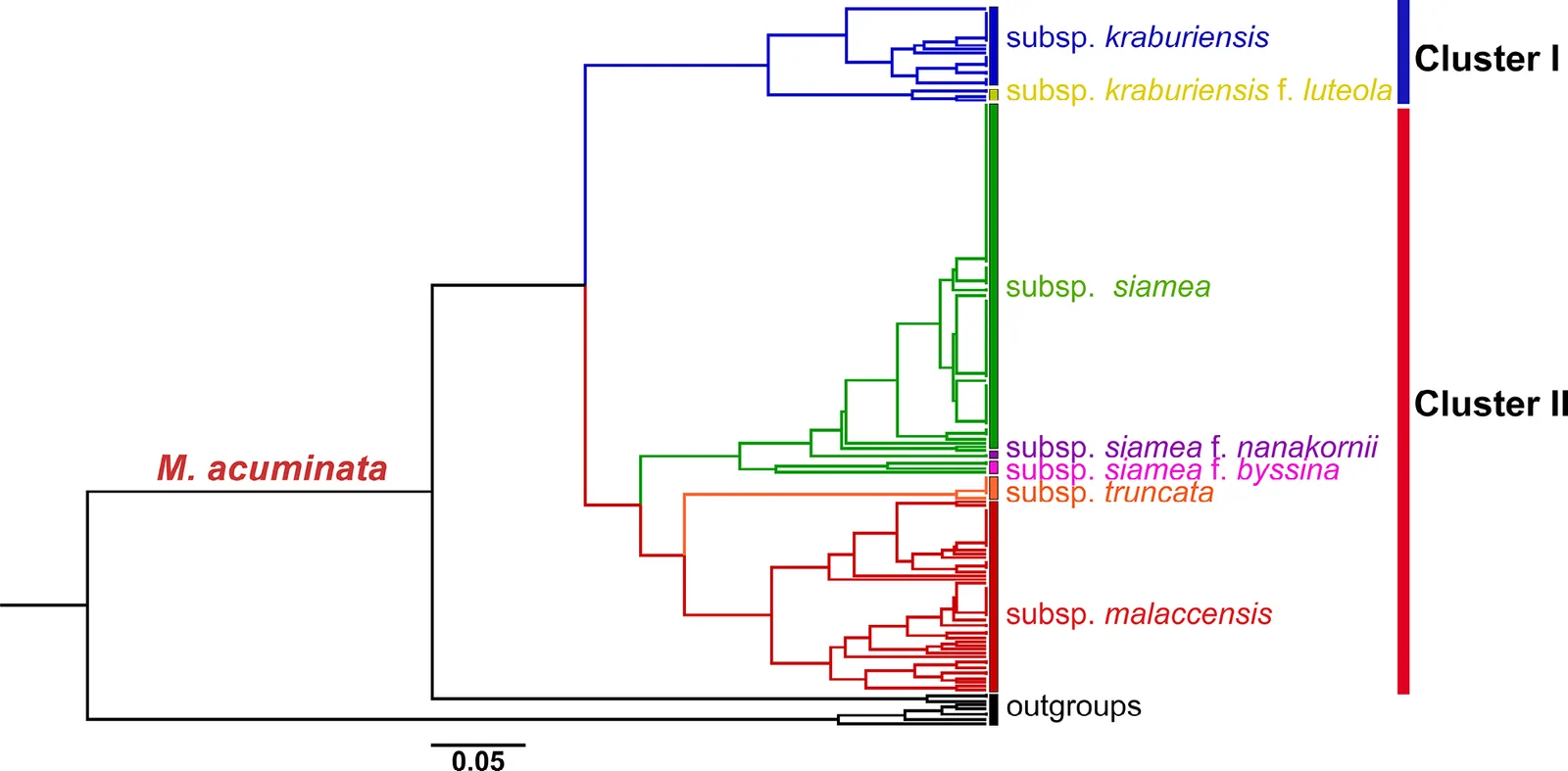
Cluster hierarchy of the 186 accessions of *Musa acuminata* and nine outgroup accessions based on 16 morphological characters, Gower’s distance, and a UPGMA clustering algorithm. Note: The final rendered hierarchical clustering displays visible tips, as closely related terminal clusters with short cluster distances were collapsed into single representative nodes to ensure readability and prevent overlapping text.

A multivariate analysis of 16 morphological characters (Table 1, Suppl. material 2) revealed several differences of diagnostic value among the four Thai subspecies of *M. acuminata* (Fig. 6). These included vegetative features such as the shape of the leaf base (Fig. 3), rachis position (Fig. 4), and shape of the male-phase inflorescence, as well as the arrangement and color of the bracts (Fig. 5). The cluster analysis revealed two major morphological clusters determined mainly by the position of the inflorescence. The first cluster (cluster I), which corresponds to the new subsp. *kraburiensis*, exhibited a horizontal to upward-curving inflorescence/infructescence rachis. The second cluster (cluster II), which comprises the accessions of the remaining three subspecies, is characterized by a downward-pointing inflorescence/infructescence rachis, ranging from curved to drooping. Within cluster II, the shape of the leaf base, bract arrangement, and color were all of diagnostic value for distinguishing among subspecies. *Musa acuminata* subsp. *siamea* or subsp. *burmannica* sensu Simmonds is characterized by cuneate to oblique leaf bases and a conspicuously imbricate arrangement of the rachis bracts. In contrast, *M. acuminata* subsp. *malaccensis* and subsp. truncata stood out by having round, cordate, or auriculate leaf bases and slightly imbricate to non-imbricate rachis bracts. Furthermore, the rachis bracts of all three subspecies constituting cluster II exhibited distinct coloration (Fig. 5). Within cluster II, subsp. truncata differs from the sympatric subsp. *malaccensis* in its unique bract coloration. In subsp. truncata, the inflorescence bracts are dark purple externally (abaxially) and purplish red or ivory internally (adaxially), sometimes with a pale red tinge. By contrast, the bracts of subsp. *malaccensis* are red, purplish red, or pinkish purple on both surfaces (Fig. 5). The UPGMA analysis of morphological characters separated the *formae* of subsp. *kraburiensis* and subsp. *siamea*. Within subsp. *kraburiensis*, f. *luteola*, characterized by its yellow bracts, formed a distinct cluster separate from f. *kraburiensis*. Although subsp. *siamea* f. *nanakornii* possesses numerous male buds, a feature uncommon among other taxa of *M. acuminata*, its cuneate to oblique leaf base supports placement within subsp. *siamea*.

### Morphological deviations in *Musa acuminata* subspecies

In addition to the known variation in the shape of the leaf base, rachis orientation, and arrangement of the apical rachis bracts in the *M. acuminata* subspecies complex, several unusual features were revealed in the wild Thai populations studied. These included yellow rachis bracts in some populations of *M. acuminata* subsp. *siamea* and subsp. *kraburiensis* and a curious deviant inflorescence morphology in some populations of *M. acuminata* subsp. *siamea*.

**Figure 7.**
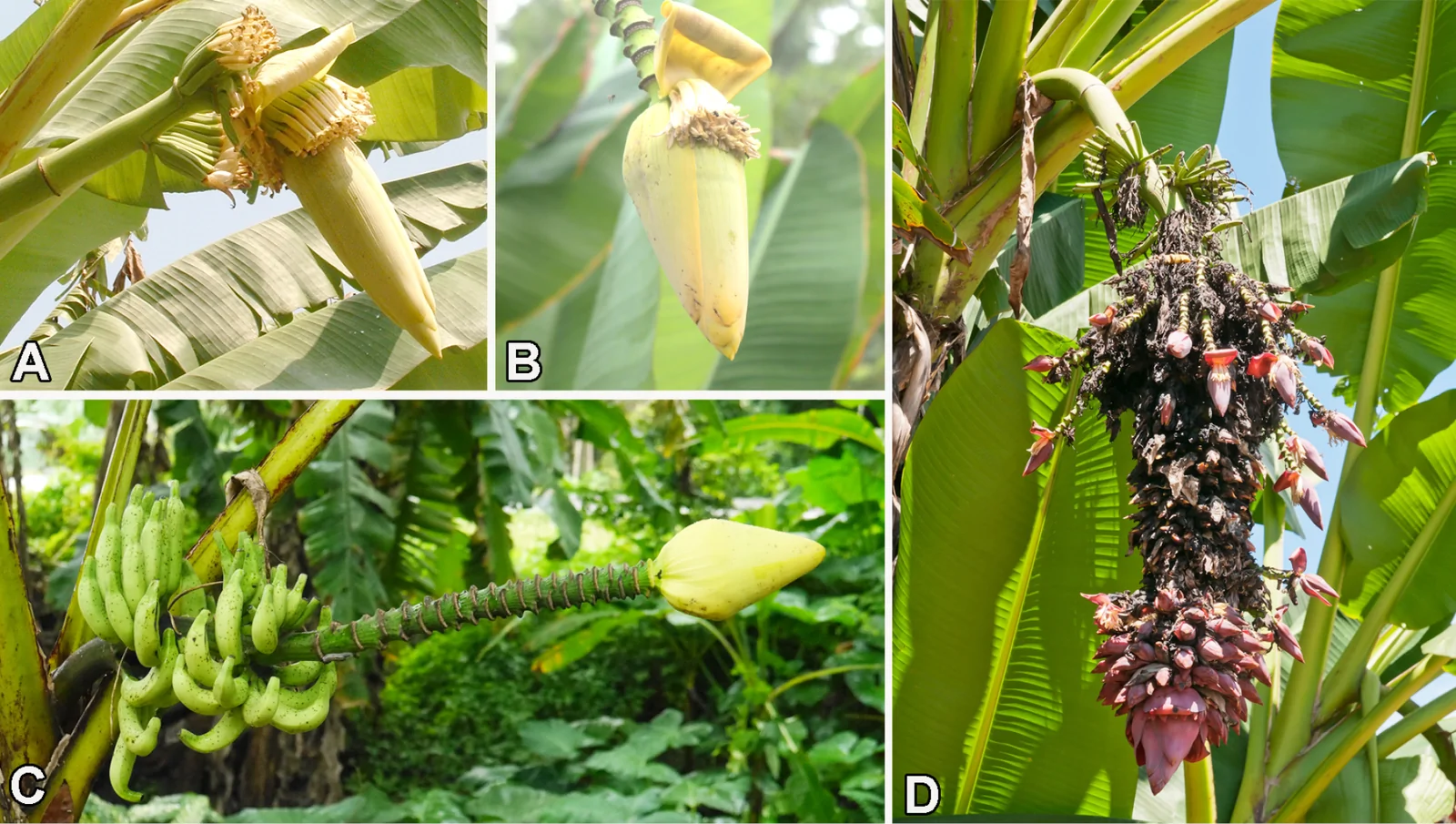
Morphological deviations in *Musa acuminata* subspecies. **A, B**. Inflorescences in the female and male phases, respectively, of the yellow-bracted mutant *M. acuminata* subsp. *siamea* f. *byssina* Swangpol & Athawongsa, f. nov., Swangpol & Somana 173, from Nakhon Ratchasima (NE Thailand); **C**. Male-phase inflorescence of the yellow-bracted mutant *M. acuminata* subsp. *kraburiensis* f. *luteola* Swangpol & W.Inta, f. nov., Swangpol & Somana 251, from Ranong (Peninsular Thailand); **D**. *M. acuminata* subsp. *siamea* f. *nanakornii* Swangpol, Chom. & Sukkaew, f. nov., showing aberrant lateral branch inflorescences during the male phase, Swangpol & Somana 448, from Nan (N Thailand). Photos by Sasivimon C. Swangpol.

A new form, f. *byssina* (Fig. 7A, B), of *M. acuminata* subsp. *siamea* was recently recorded in several locations in Chanthaburi (SE Thailand), Nakhon Ratchasima (E Thailand), and Kamphaeng Phet (N Thailand). It stands out by having yellow rachis bracts, in contrast to the typical red bracts of subsp. *siamea*. In Ranong (Peninsular Thailand), a new yellow-bracted mutant population of *M. acuminata* subsp. *kraburiensis*, named here f. *luteola* (Fig. 7C), was observed. It grew intermixed in a population of c. 50 individuals of the typical red-purple form scattered over approximately 4 km^2^ of a large open area. Animal visitation was observed in both the mutant and wild-type forms (Nilapaka, pers. obs.). Reduced levels or the absence of flavonols and anthocyanins have been reported in f. *luteola*, probably due to mutations in the genes encoding flavonol synthase, dihydroflavonol reductase, or other related enzymes in these pathways (Kitdamrongsont et al. 2008; Pothavorn et al. 2010). The yellow-bracted mutants are particularly common in some areas. This is probably a consequence of genetic drift and inbreeding within a critically small panmictic population due to deforestation and forest fragmentation.

**Figure 8.**
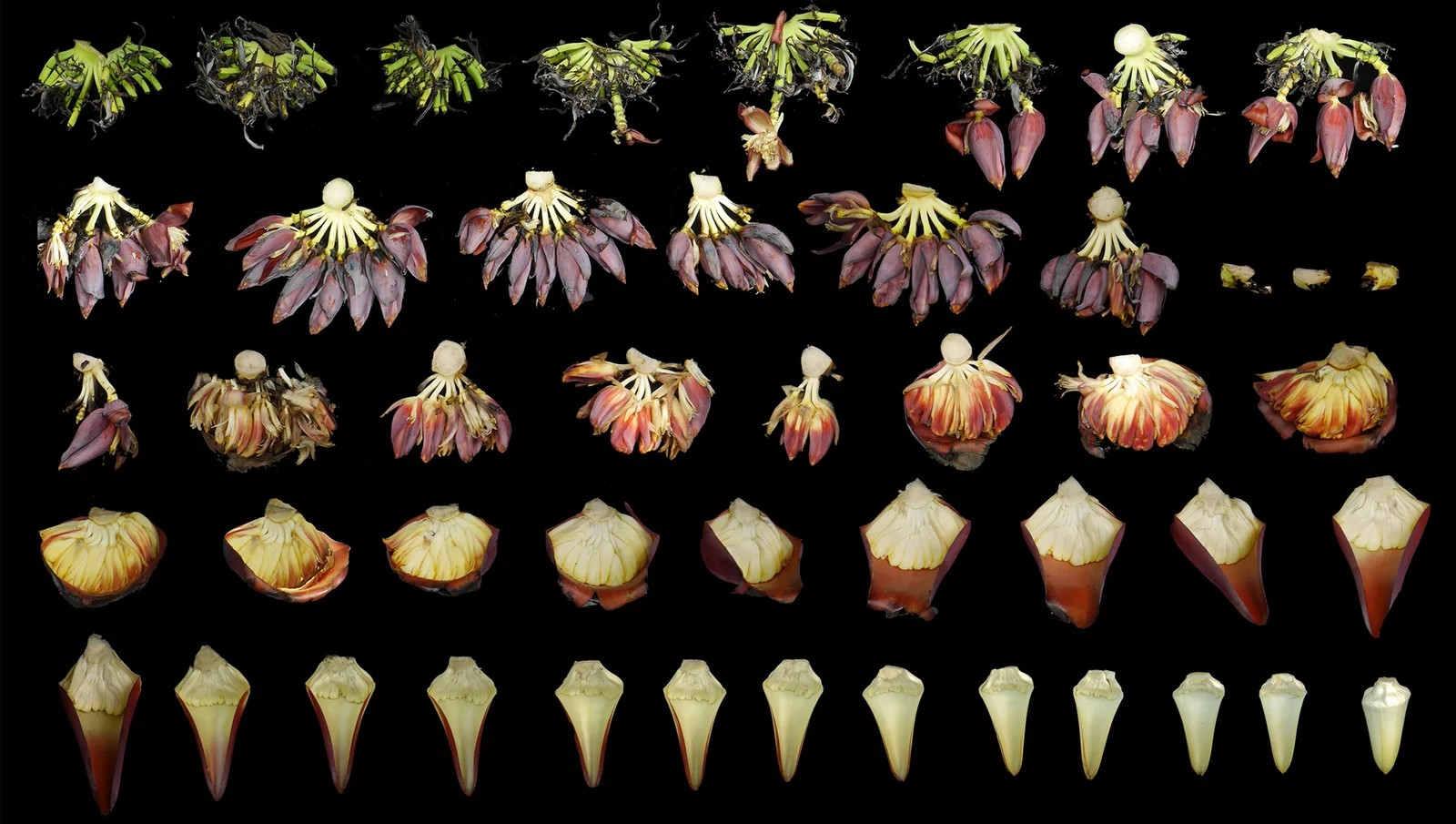
Developmental series of hands in the inflorescence of *Musa acuminata* subsp. *siamea* f. *nanakornii* (‘Kluai Roi Pli’). Images are arranged from older to younger developmental stages, proceeding from the upper left to the lower right. The uppermost left image represents a hand of female flowers. The subsequent hands show the abnormal male flowers, in which individual flowers are replaced by secondary male-phase inflorescences with various degrees of abnormality. This specific specimen, grown in Ratchaburi, Thailand, lacked an extended rachilla. Photos by Sasivimon C. Swangpol.

An anomaly in the inflorescence architecture was observed in a mutant form of *M. acuminata* subsp. *siamea*, which is described here as forma *nanakornii* (Figs 7D, 8, 10). It is cultivated by several Hmong hill-tribe communities in the highlands of N and NE Thailand. The Thai name, ‘Roi Pli,’ refers to the occurrence of numerous lateral male-phase inflorescences produced on the main inflorescence axis. The unusual chandelier-like appearance of the inflorescence is caused by the primordia of the cincinni producing inflorescences instead of male flowers. Although this forma typically does not produce seeded fruit, an accession bearing a few seeded fruits has been observed (Swangpol & Somana 558).

## Discussion

Previous studies of genetic diversity within the *M. acuminata* complex have suggested geographic distribution as a determining factor (Cheesman 1947, 1948; Simmonds 1956; De Langhe et al. 2000; Perrier et al. 2009, 2011; Jenny et al. 2024). However, delimitation of wild *Musa* species is difficult. The herbaceous habit and large dimensions of the leaves and inflorescences of bananas make the preservation of herbarium specimens challenging (e.g., Simmonds 1956; De Langhe et al. 2000). Many specimens include only immature fruits, which are insufficient for correct identification. Published records often provide insufficient descriptions that focus mainly on pseudostem and inflorescence characteristics, rendering identification problematic. This study aims to address these limitations through repeated field campaigns in Thailand, providing detailed documentation of morphological and genetic variation. All voucher specimens were prepared from plants occurring in the wild and were georeferenced and photographed. The colors were described using a standard color chart (IPGRI-INIBAP/CIRAD 1996). Representatives of the selected taxonomic entities were sketched in the field and later inked for the final plates.

### Taxonomic status of the *M. acuminata* complex

Segregation of the *M. acuminata* complex in Thailand into species and subspecies corroborated previous taxonomic treatments (Simmonds 1956; De Langhe et al. 2000; Wong et al. 2001; Perrier et al. 2009; Rouard et al. 2018). Dendrograms generated from morphological and distributional data (Fig. 6) strongly supported the recognition of *M. acuminata* as a distinct species, consistent with conventional classifications (Cheesman 1948; Simmonds 1956; Nasution 1991; Wong et al. 2001; Inta et al. 2023). The extensive sampling of *M. acuminata* across Thailand provides substantial evidence supporting the use of subspecific ranks within the complex, a view contrasting with Nasution’s (1991) classification of all *M. acuminata* variations in Indonesia as varieties. Nasution erected 15 varieties to accommodate the subspecific variation, including nine new combinations encompassing *acuminata*, *malaccensis*, and *microcarpa*, but did not provide justification for these taxonomic decisions. The findings align more closely with Simmonds’ (1956: 469) assertion that the various forms of *M. acuminata* represent “distinct geographico-morphological units.” Indeed, the geographically defined distributions of the *M. acuminata* infraspecific taxa observed in this study are congruent with the results of phenetic analyses (Perrier et al. 2009).

Moreover, the status of the four subspecies—*siamea*, *malaccensis*, *truncata*, and the newly described *kraburiensis*—is well supported by the morphological analysis (Fig. 6). The separation of the four subspecies, as shown in the following key, was determined mainly by leaf base shape, rachis position, bud imbrication, and the color of male bracts. Meanwhile, several characters previously used by banana researchers were found to be highly variable among different populations within the subspecies. These ambiguous characters included blotches on the leaves of young suckers, pseudostem pigmentation, and waxiness of the leaf sheaths. In addition, the shapes of the free tepals used by Nasution (1991) to discriminate var. *malaccensis* and var. *microcarpa* were not helpful. Nasution described var. *malaccensis* as having a rounded, acuminate free tepal and var. *microcarpa* as having an obovate, acute free tepal. Previous work (Inta et al. 2023) examined floral morphological evolution in *Musa* and suggested that tepal morphology is uniform across *M. acuminata* subspecies, with the tepals of all subspecies exhibiting an elliptic shape and an acuminate apex.

### On the identity of subspecies *siamea* and *burmannica*

Although *M. acuminata* subsp. *siamea* and subsp. *burmannica* (mentioned as the ‘Annam’ and ‘Tavoy’ forms, respectively, by Cheesman 1948) were traditionally described as having similar obtuse, imbricate, and red-purple male-phase inflorescences, they were previously separated based on waxiness and pigmentation of the pseudostems, bud shapes, bunch position and compactness, and the length of the fruit pedicel and apex (Cheesman 1948; Simmonds 1956; De Langhe and Devreux 1960). The collection lacked accessions exhibiting the complete suite of characteristic traits of Simmonds’ subsp. *siamea* (waxy pseudostem, subhorizontal and compact bunch, tepals often with yellow tips, and polygonal-round fruit cross section) or the corresponding suite of traits of Simmonds’ subsp. *burmannica* (non-waxy pseudostem, pendant and lax bunch, and angular fruit cross section).

Further confusion arises from the use of fruit pedicel and apex lengths, which different authors have ascribed to the subspecies. Cheesman (1948: 27) described the “Tavoy form” (*M. acuminata* subsp. *burmannica* sensu Simmonds) as having “the pedicel very short and not very distinct, acumen approximately 0.5 cm long.” Conversely, he described the “Annam form” (*M. acuminata* subsp. *siamea* sensu Simmonds) as having “a short (0.5 cm) but quite distinct pedicel and a well-marked acumen 0.5 cm long.” However, De Langhe and Devreux (1960) presented contrasting data in their comparative morphological table. They stated that subsp. *burmannica* has a long fruit pedicel (1 cm) and a long apex (1 cm or longer), whereas subsp. *siamea* has a shorter fruit pedicel and apex (0.5 cm).

Jenny et al. (2024) also reported that the morphological traits they examined were insufficient to definitively classify specimens within the subsp. *siamea*/*burmannica* complex because of the morphological similarity of these specimens.

Cheesman (1948), Simmonds (1962), and Perrier et al. (2009) reported that subspecies *burmannica* and *siamea* are distributed in overlapping areas from southeastern India and Myanmar to Vietnam. In these cases, it is suspected that the reported banana collections were too limited to be definitive. Cheesman (1948: 22) himself noted observing only a “dozen clones” of *M. acuminata* in the I.C.T.A. collection. Therefore, despite extensive surveys along the Thailand–Myanmar border from Mae Hong Son through Prachuap Khiri Khan, no “true” subsp. *burmannica* accession *sensu* Simmonds was found, and subsp. *burmannica* cannot be maintained in the traditional sense. It is argued that subsp. *burmannica* should be included within subsp. *siamea*, which possesses cuneate or oblique leaf bases; purple, reddish-purple to purplish-brown male-phase inflorescences; variable degrees of bract imbrication; and bract apices overlapping by more than 1 cm.

This argument is supported by the results of Diversity Arrays Technology (DArT), simple sequence repeat (SSR) markers, molecular cytogenetics, and genotyping-by-sequencing (GBS), which indicated that the two subspecies were not separated (Dupouy et al. 2019; Jenny et al. 2024).

According to the rules of botanical nomenclature (Turland et al. 2018), among the two subspecies proposed by Simmonds (1956), subsp. *siamea* takes precedence because it appeared earlier (page 466). Therefore, subsp. *siamea* is the accepted name, with subsp. *burmannica* (page 468) reduced to synonymy.

### *M. acuminata* complex of subsp. *microcarpa* and subsp. truncata

Simmonds (1956) distinguished subsp. *microcarpa* from its counterparts “by the yellowish tinge and virtual waxlessness of the foliage; by the intense chocolate brown pigmentation of sheaths and, often, midribs; by the fading purple flush on the peduncle and male(-phase) rachis; by the plump non-imbricate male bud (male-phase inflorescence with bract apices not overlapping); by the bracts purple without and pale red within but weakly rolled at the time of falling; and by its essentially montane distribution. The fruits are variable in size.” All these morphological features, especially the male-phase inflorescence features (Fig. 5), are variably present in subsp. *truncata*, subsp. *malaccensis*, and subsp. *siamea* found in Thailand and could not be used for identification.

Simmonds (1956) reduced *M. truncata* to a synonym of *M. acuminata* subsp. *microcarpa* because of their similar morphology. However, AFLP and RFLP marker analyses recently revealed their distinct status (Carreel et al. 1994; Wong et al. 2001; Perrier et al. 2009). Previous literature stated that subsp. *microcarpa* was endemic to the lowlands of the island of Borneo (Nasution 1991; Wong et al. 2001) and that subsp. *truncata* is distributed in the highlands of Peninsular Malaysia (Carreel et al. 1994; Perrier et al. 2009). The fact that subsp. *microcarpa*, according to Simmonds’ description, could not be found in Thailand agrees with its geographic distribution and the genome assemblies recently reported by Perrier et al. (2011) and Rouard et al. (2018). Cheesman (1948) also mentioned that subsp. *truncata* plants were not vigorous under less humid and lowland conditions and ultimately died. It is suspected that geographic isolation may have caused subspecific polymorphisms between the two subspecies.

### Distribution boundaries of *M. acuminata* subspecies

The survey results confirmed Simmonds’ (1956) view that Peninsular Thailand and the Thailand–Myanmar border are areas of “extreme complexity.” *Musa acuminata* subsp. *truncata*, as a new record for Thailand, extends its known natural distribution from mountainous areas of mainland Malaysia across the Thailand–Malaysia border. The results agree with earlier reports (Cheesman 1948; Simmonds 1956; Wong et al. 2001) that subsp. *truncata* is montane and does not thrive well at low altitudes, as the sole accession of this subspecies could not be maintained in the nursery near Bangkok.

*Musa acuminata* subsp. *siamea* is more drought- and heat-tolerant and is the most widespread of all the subspecies. It was found throughout northern, central, western, northeastern, and eastern Thailand, where a dry season of 3–6 months occurs. Evidence from other reports indicates that this subspecies has also been observed in southern China, Laos, and Vietnam (Wu and Kress 2000; Lheureux et al. 2007; Jenny et al. 2024). The absence of *M. acuminata* specimens from most of northeastern and eastern Thailand (Fig. 1) may be due to extensive and continuous deforestation in these regions. Meanwhile, subsp. *microcarpa*, which has not been found in Thailand, is probably restricted to the island of Borneo (Perrier et al. 2011; Rouard et al. 2018). However, field surveys of subsp. *microcarpa* in its natural habitats are needed to confirm its existence or its synonymy with subsp. *siamea*.

Based on the investigations, the distribution of *M. acuminata* subsp. *malaccensis* has been extended from southern Peninsular Malaysia to northwestern Thailand. This subspecies prefers cool conditions and continuous precipitation throughout the year. The morphological analysis did not support the assumption of De Langhe et al. (2000) that these northern populations were ‘pseudo-malaccensis.’ In contrast, it is believed that they are typical of their southern counterparts, and it is hypothesized that this subspecies might have been distributed along the mountain chain, which had the same climate pattern from south to north, since ancient times when the climate was colder and rainfall was higher. When the climate became warmer and drier, the distribution range receded. Some populations have persisted in isolated areas where the climate has remained wet and cool. Exploration along the rainward side of the Tanao Si/Tenasserim mountain chain in Myanmar would be important to provide more evidence for this hypothesis. Subspecies *malaccensis* was commonly found in the lowlands of southern Thailand and also grew in areas overlapping with populations of subsp. *truncata* in the highlands of Narathiwat and Yala. The fact that it was found at approximately 200–1,000 m above sea level in northern Thailand leads to the conclusion that it has a much wider altitudinal range than previously reported (Ridley 1924; Simmonds 1956; Nasution 1991; Wong et al. 2001; Perrier et al. 2009).

### Deviation of *M. acuminata* subspecific forms

Three new taxonomic entities of *M. acuminata* are reported in this manuscript (Fig. 7): two forms with yellow bracts and one with a strikingly deviant inflorescence morphology. Many *Musa* taxa have been listed as having yellow bracts, e.g., *M. basjoo* Siebold ex Miq. (Miquel 1867), *M. siamensis* Häkkinen & Rich.H.Wallace (Häkkinen and Wallace 2007), *M. acuminata* var. *flava* (Ridl.) Nasution (Nasution 1991), and *M. acuminata* subsp. *banksii* (F.Muell.) N.W.Simmonds (Simmonds 1956). Forma *byssina* and forma *luteola*, described here, belong to *M. acuminata* subsp. *siamea* and subsp. *kraburiensis*, respectively. They are easily distinguished from *M. acuminata* var. *flava*, a native variety distributed in Kalimantan and on the Malay Peninsula, by lacking a cuneate or oblique leaf base and a convolute male-phase inflorescence. The name *byssina* refers to the bracts having a light yellow color reminiscent of raw silk. The name *luteola* denotes the golden-yellow color of egg yolk (Eckel 2010–2023).

In their natural habitats, f. *byssina* appeared to be less common than f. *luteola*. However, it may not actually be less common. The area south of Phanom Dongrek Mountain has many yellow-bracted populations, but they are patchily distributed over large areas and can grow only where there is sufficient moisture and limited human disturbance. Despite being observed in only one area of the Isthmus of Kra, the f. *luteola* population extended over 4 km, and the number of clumps reached up to 50. In contrast, f. *byssina* was represented by only a single clump at each of several locations among typical red-purple *M. acuminata* populations. Nevertheless, preliminary observations showed squirrels and bats visiting the yellow flowers of f. *luteola* at night and insects visiting during the day (W. Nilapaka, pers. obs.). The lighter bract color may attract these nocturnal and diurnal visitors. Bract color may be less important for pollinator attraction than floral scent and nectar or pollen rewards.

The extraordinary inflorescence architecture of *M. acuminata* subsp. *siamea* f. *nanakornii* (Fig. 7D) has attracted plant enthusiasts and researchers alike. Because of the infertility of the form, the three initial specimens collected were vegetatively propagated and grown by the villagers as ornamental plants. For research purposes, a clone found in Chiang Mai was introduced into the Queen Sirikit Botanic Garden, and Chomchalow et al. (2014) reported these bunch mutations for the first time. The aberration, in which a male-phase inflorescence replaces each flower on the hands of the banana bunch, is possibly caused by abnormalities involving flower and inflorescence meristem indeterminacy. This mutation might involve the abnormal expression of a transcription factor or other genes in related pathways. The genetic and hormonal regulation of inflorescence meristem activity has been reviewed in angiosperms (Thompson and Hake 2009) and studied in monocots, e.g., corn (Laudencia-Chingcuanco and Hake 2002; Chuck et al. 2008) and grasses (Bommert and Whipple 2018), and in dicots, e.g., cauliflower (Azpeitia et al. 2021). However, many studies of the genome, transcriptome, and cell and tissue differentiation are required because almost no studies of inflorescence formation in bananas have previously been conducted.

There are five main conclusions from this study. First, *M. acuminata* is discontinuous from the other related species investigated. Second, the use of subspecific rank within the *M. acuminata* complex is considered the most suitable way of dealing with the variation encountered in the wide-ranging collections of bananas throughout Thailand. Third, subsp. *microcarpa* has not been found in Thailand, and subsp. *burmannica* cannot be maintained and is reduced to synonymy with subsp. *siamea*. Fourth, the addition of subsp. *kraburiensis* as a newly described subspecies and subsp. *truncata* as a new record for the Thai flora brings the total number of Thai subspecies to four. Lastly, the taxonomic status of these four *M. acuminata* subspecies—*kraburiensis*, *malaccensis*, *siamea*, and *truncata*—found in Thailand is supported by morphology.

Based on this study, in addition to geographic distribution, the most important morphological characters for the determination of these *M. acuminata* subspecies are leaf base shape, rachis position, male-phase bract imbrication, and bract color. The minimal collection requirements for accurate identification are the base of one mature leaf, a male-phase inflorescence, and a hand of fully mature fruits. In addition, four series of photographs are needed: (1) the clump of pseudostems with a mature bunch showing the rachis position; (2) the leaf base and apex on their adaxial sides and the middle part of the leaf on its abaxial side to represent the waxiness and color of the underside; (3) the male-phase inflorescence and outermost bracts showing their shapes, external and internal bract colors, and reflexing after anthesis; and (4) the male flowers showing the adaxial side of the compound tepal, free tepal, stamens, and pistil. The standard color chart (e.g., IPGRI-INIBAP/CIRAD 1996) and a scale should always accompany all photographs.

For further studies of genetic variation, mixing among subspecies, and the actual natural distribution of *M. acuminata* subpopulations in relation to geographic locations, multilocus genetic assays such as SSR, SNP, or inter-MITE analyses are required.

### Taxonomic treatment

#### M. acuminata

Taxon classificationPlantaeZingiberalesMusaceae

Colla, Mem. Gen. Musa 25: 394. 1820

7EBA254C-6B34-5709-9497-6C9B3BDC7CEA

##### Diagnosis.

*Musa acuminata* is distinguished from *M. balbisiana* by its revolute bracts, bare rachis, and non-clasping petiole canal margins, two rows of ovules in one locule; in contrast, *M. balbisiana* possesses imbricate bracts, a rachis with persistent bracts, and clasping petiole canal margins, four rows of ovules in one locule.

##### Type.

Rumphius (1747), Herbarium Amboinense 5: t. 61 Fig. 1, *M. simiarum* Pissang Jacki. (lectotype: designated by Häkkinen and Väre 2008).

##### Description.

***Pseudostem*** of main shoot 1.4–7 m tall, 17–65 cm in circumference, light green, greenish yellow to medium green, with 1–6 suckers, often with large blotches. ***Leaf*** blade oblong, 80–340 by 25–95 cm, with or without blotches, with thin to thick wax cover, base cuneate or rounded to auriculate, more or less oblique, apically blunt; petiole 16–130 cm long, proximally with dark purple-brown blotches varying in size, margins inward curved to spreading. ***Peduncle*** glabrous, or covered by short hairs varying in density. ***Inflorescence*** curved or drooping; rachis bract lanceolate to elliptical or ovate, varying in color on the abaxial side from dark purple to orange-red, sometimes with yellow striation, revolute before falling. ***Flowers*** with ivory-colored perianth and yellow petals lobes, sometimes with pink tinge; female flowers with an ovate, inner adaxial tepal, green ovary, and ivory-colored stigma; male flowers with an elliptic free adaxial inner tepal, ivory-colored ovary, and yellow to orange stigma. ***Infructescence*** c. 120 cm long, drooping; peduncle 30–75 cm long, rounded in cross section and c. 4 cm across; 3–15 hands per bunch, 5–23 fruits per hand; biseriate male-phase rachis bracts not persistent after anthesis. ***Fruits*** inserted in two rows; pedicel 0.3–2 cm long; berry 5–12 long cm by 1.5–2.5 cm wide; apex bottle-shaped, 0.4–1.5 cm. ***Seeds*** brown to black, irregular in size and shape, angular in cross section, with rough surface.

##### Distribution.

India (Assam), Myanmar, Thailand, Laos, Vietnam, China, Malaysia, Indonesia (type), and Australia (Queensland).

##### Notes.

The most widely distributed species occurring throughout Thailand. Four subspecies are recognized here. Transitional forms have been observed in areas of overlapping distribution, suggesting possible hybrid zones.

### Key to subspecies of *M. acuminata*

**Table:** 

1	Leaf base cuneate to oblique, bract apex overlapping 1–2 cm	**2**
–	Leaf base round, cordate or auriculate, bract apex not overlapping or overlapping less than 1 cm	**4**
2	Bracts purple, bluish purple to purple brown	**3**
–	Bracts yellow	***M. acuminata* subsp. *siamea* f. *byssina***
3	Male-phase inflorescence on one rachis one	***M. acuminata* subsp. *siamea* f. *siamea***
–	Male-phase inflorescences on one rachis numerous	***M. acuminata* subsp. *siamea* f. *nanakornii***
4	Peduncle and rachis purplish brown and dark purple, external bracts dark purple, internal bract ivory to pale red-purple fading to ivory at the base	***M. acuminata* subsp. truncata**
–	Peduncle and rachis green, external and internal bracts purple, red-purple, red to orange red, rarely yellow	**5**
5	Rachis 2.5 cm or less in diameter, horizontal to pendulous; male-phase inflorescence apex directed downward	***M. acuminata* subsp. *malaccensis***
–	Rachis more than 3.0 cm in diameter, horizontal to ascending; male-phase inflorescence apex directed horizontally or upward	**6**
6	Bract red, orange-red, red purple or pink-purple	***M. acuminata* subsp. *kraburiensis* f. *kraburiensis***
–	Bract yellow	***M. acuminata* subsp. *kraburiensis* f. *luteola***

#### Musa acuminata Colla subsp. kraburiensis

Taxon classificationPlantaeZingiberalesMusaceae

Swangpol & Somana subsp. nov.

F1EDC7AB-0E39-5BDC-B3D5-31BCDBEE306C

urn:lsid:ipni.org:names:77394109-1

Fig. 9

##### Diagnosis.

*Musa acuminata* subsp. *kraburiensis* is distinguished from *M. acuminata* subsp. *malaccensis*, *M. acuminata* subsp. *siamea* and *M. acuminata* subsp. *truncata* by horizontal or angled upward rachis, rachis diameter more than 2.5 cm and male-phase inflorescence angled upwards.

##### Type.

Thailand: • Kapoe District, Ranong Province, 15 December 2023, with flowers, *Swangpol & Somana 689* (holotype: BKF!).

##### Description.

***Pseudostem*** of main shoot 1.7–4.0 m tall, 25–50 cm in circumference, light green, with 2–4 suckers. ***Leaf*** petiole 40–100 cm long, with small to large dark purple-brown to brown blotches, petiolar canal straight with erect margins; leaf blade 105–250 cm by 40–95 cm, with little or no visible sign of wax; basally rounded, cordate or auriculate. ***Inflorescence*** peduncle 15–40 cm long, sometimes with short hairs, in the female phase fusiform to conical in outline, 20–40 cm by 10–15 cm; in the male phase lanceolate to ellipsoid to ovate in outline, with apices of the inner rachis bracts completely concealed by the outer bracts; both sides of bracts covered by thin layer of wax, purple, red-purple, pink-purple to orange-red, rarely yellow, sometimes with yellow or red purple streaks on the abaxial side; bract revolute before falling. ***Rachis*** at anthesis horizontal or angled upwards, rachis diameter more than 3 cm, green, bracts leaving prominent scars after shedding. ***Flowers*** with ivory-colored perianth and yellow petals lobes; female flowers with an ovate, inner adaxial tepal, green ovary, and ivory-colored stigma; male flowers with an elliptic free adaxial inner tepal, ivory-colored ovary, and yellow to orange stigma. Infructescence. ***Infructescence*** horizontal, with 4–15 hands per bunch and 5–24 fruits per hand. ***Fruit*** pedicel 0.5–1.0 cm long; berry 2–10 cm by 1.3–3.0 cm; apex bottle-necked, 0.5–1.5 cm. ***Seeds*** brown to black, angular in cross section, with rough surface.

**Figure 9.**
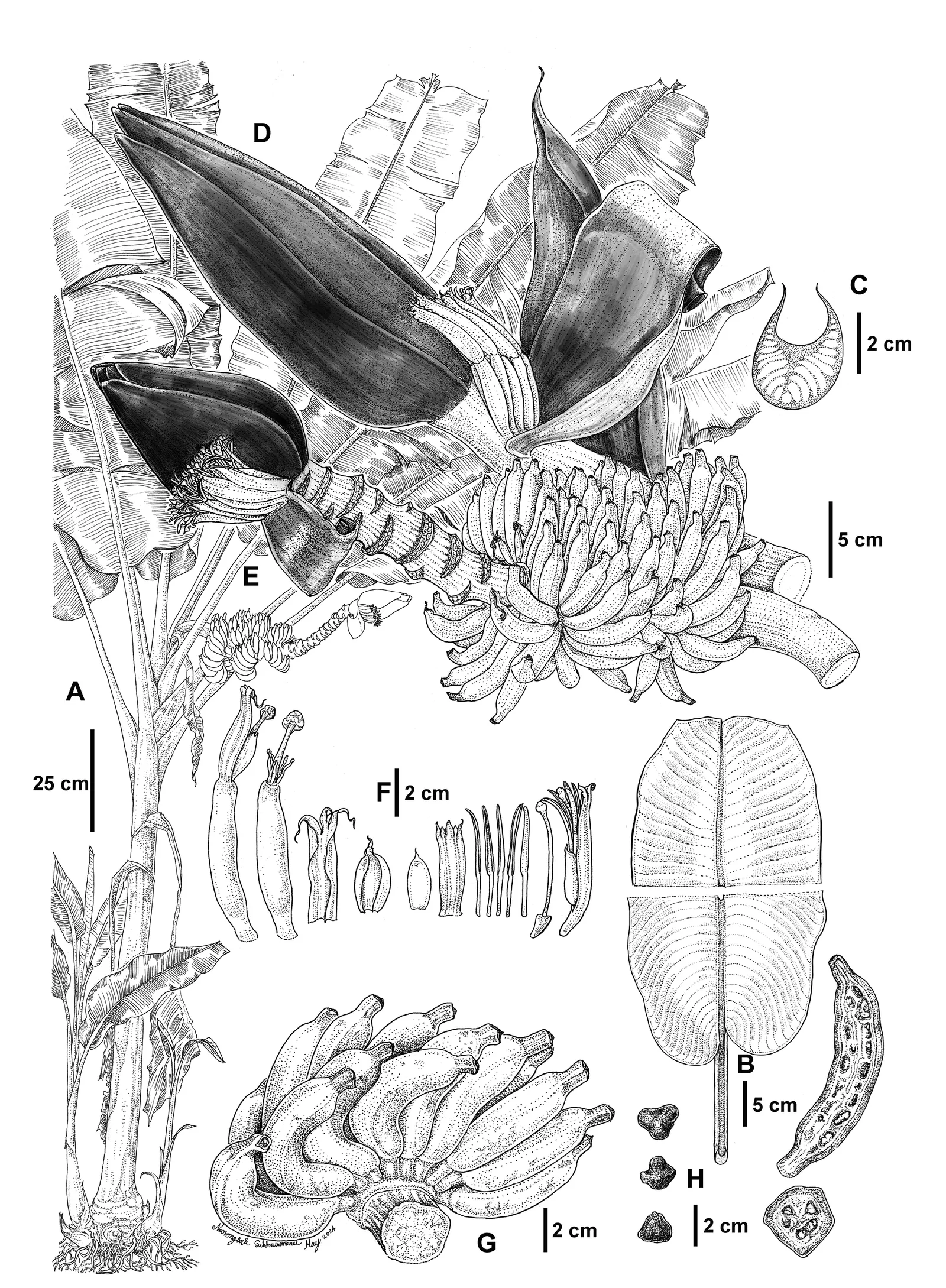
*Musa acuminata* subsp. *kraburiensis* Swangpol & Somana, subsp. nov. **A**. Clump; **B**. A leaf showing the base and apex; **C**. A transverse section of the petiole; **D**. A female-phase inflorescence; **E**. A male-phase inflorescence; **F**. Flowers showing, from left to right, a female flower, a female flower without the tepals, a compound tepal and a free tepal of a female flower, a free tepal and a compound tepal of a male flower, five anthers, a pistil, and a male flower; **G**. Left, a hand of fruits, and right, transverse and longitudinal sections of fruits. Illustrated by Narongsak Sukkaewmanee; graphic by Sasivimon C. Swangpol and Wandee Inta.

##### Distribution and habitat.

Endemic to the Kra Isthmus, at ca. 9–10.5°N, in Peninsular Thailand along the Tanao Si/Tenasserim mountain chain. The northern border of the distribution area extends from Pathio to Phato District, Chumphon Province, and ends in Suk Samran District, Ranong Province.

##### Ecology.

Scatter populations of this subspecies are found on hills, slopes, and open areas near streams along Routes No. 4 and No. 4006 and in fruit orchards in Amphoe Lang Suan.

##### Etymology.

The epithet *kraburiensis* refers to the Kraburi River basin in the vicinity of the Kra Isthmus, where this taxon is endemic.

##### Vernacular.

Kluai Pa Kraburi (Thai: กล้วยป่ากระบุรี, “wild banana from Kraburi”).

##### Notes.

The Kra Isthmus area where subsp. *kraburiensis* was found is in the biogeographic transition zone between two floristic provinces, Indochinese and Sundaic (Woodruff 2003). The subspecies is possibly a natural fertile hybrid connecting subsp. *siamea* at the southernmost end of its distribution boundary with the northernmost area of subsp. *malaccensis* in Peninsular Thailand. At the boundary in Amphoe Tha-Sae, Changwat Chumphon, there are also subsp. *siamea*–subsp. *kraburiensis* hybrid-like plants. Meanwhile, in Amphoe Lang Suan and Amphoe Phato, Changwat Chumphon, subsp. *kraburiensis* grows alongside subsp. *malaccensis*. A yellow-bracted form of this subspecies is found in Changwat Ranong.

#### M. acuminata Colla subsp. kraburiensis

Taxon classificationPlantaeZingiberalesMusaceae

Swangpol & Somana f. luteola Swangpol & W.Inta f. nov.

86C3527D-751F-53FD-AA7A-56D3BC11FFF7

##### Diagnosis.

*Musa acuminata* subsp. *kraburiensis* f. *luteola* is distinguished from f. *kraburiensis* by its yellow bracts, whereas the male bracts of f. *kraburiensis* are typically red to orange-red, or occasionally red-purple to pink-purple.

##### Type.

Thailand: • Kapoe District, Ranong Province, 15 December 2023, with male-phase inflorescence, Swangpol & Somana 690 (holotype: BKF!); • Mueang Ranong District, Ranong Province, 24 September 2016, with male-phase inflorescence, Swangpol & Somana 601 (paratype: BKF!).

##### Description.

***Pseudostem*** of main shoot 2–3.5 m tall, 20–45 cm in circumference, light green, with 2–4 suckers. ***Leaf*** petiole 60–80 cm long, without blotches or with small to large dark brown blotches, petiolar canal straight with erect margins; leaf blade 140–250 cm by 50–80 cm, with little or no visible sign of wax; basally rounded, cordate or auriculate. ***Inflorescence*** peduncle 15–35 cm long, sometimes with short hairs, in the female phase fusiform to conical in outline, 20–40 cm by 10–15 cm; in the male phase lanceolate to ellipsoid to ovate in outline, with apices of the inner rachis bracts completely concealed by the outer bracts; both sides of bracts covered by thin layer of wax, yellow; bract revolute before falling. ***Rachis*** at anthesis horizontal or angled upwards, rachis diameter more than 3 cm, green. ***Flowers*** with ivory-colored perianth and yellow petals lobes; female flowers with an ovate, inner adaxial tepal, green ovary, and ivory-colored stigma; male flowers with an elliptic free adaxial inner tepal, ivory-colored ovary, and yellow to orange stigma. ***Infructescence*** horizontal, with 5–10 hands per bunch and 5–20 fruits per hand. ***Fruit*** pedicel 0.5–1.0 cm long; berry 6–7.5 cm by 1.7–2.0 cm; apex bottle-necked, 0.5–1.5 cm. ***Seeds*** brown to black, angular in cross section, with rough surface.

##### Distribution and habitat.

Endemic to a single location in Ranong Province, Thailand. It is estimated that no more than 30 clumps of this yellow-bracted forma grow sparsely on a hillside in an area of approximately 10 km^2^.

##### Vernacular.

Kluai Pa Kraburi Pli Lueang (“wild banana from Kraburi with yellow buds” –in Thai), Kluai Pa Kraburi Si Thong (“golden wild banana from Kraburi” –in Thai).

##### Notes.

The plant is possibly a natural mutant of *M. acuminata* subsp. *kraburiensis*. Observations indicated that the yellow-bracted forma attracts squirrels, birds, and insects similarly to the red-bracted counterpart; however, the former appears intolerant of strong sunlight, weaker, and less numerous than the latter. The population is highly threatened by land clearing for agricultural purposes and road expansion.

#### Musa acuminata Colla subsp. malaccensis

Taxon classificationPlantaeZingiberalesMusaceae

(Ridl.) N.W.Simmonds, Kew Bull. 11, 3, 1956: 463–489

8AD39ABC-0D3C-5E29-B65D-D433557B8434

##### Type.

Malaysia [Selangor, Pahang], 1891, Henry Nicholas Ridley (lectotype: SING 062891).

##### Distribution and habitat.

**Thailand** • Northern: Tak (Doi Musoe, Along the Road Nos. 105 and 1090, Namtok Pa Wai), Mae Hong Son (Ban Mae Sam Lap); • Peninsular: Surat Thani (Surat Thani Rubber Research Station, Along the Road No. 401), Phangnga (Lam Pakarang, Khao Lak-Lam Ru NP, Along the Road No. 4), Phuket (Along the Road No. 402), Krabi (Wat Tham Suea, Khao Phanom Bencha Forest Park, Tha Pom Klong Song Nam), Nakhon Si Thammarat (Along the Road No. 401, Namtok Phrom Lok), Phatthalung (Khao Pu – Khao Ya NP, Khao Chai Son), Trang (Namtok Sai Rung), Satun (Namtok Than Plio, Thale Ban NP), Songkhla (Along the Road No. 42, Namtok Ton Nga Chang, Khao Kho Hong), Pattani (Namtok Mai Khao), Yala (Namtok Sukthalai, Betong Hospital, Along the Road No. 401), Narathiwat (Hala-Bala Wildlife Sanctuary).

##### Ecology.

Open areas at the edge of the forest, from 10–780 m a.s.l.

##### Vernacular.

Kluai Thuean and Kluai Pa, both mean “wild banana” –in Thai, Kluai Pa Malaka (“wild banana from Malacca” –in Thai).

##### Notes.

This subspecies is widespread throughout Peninsular Thailand and has recently been documented in new locations within the northwestern provinces but is absent from the southwestern and central areas. It is expected to occur across the border in Myanmar on the western side of the Tenasserim Range.

#### Musa acuminata Colla subsp. siamea

Taxon classificationPlantaeZingiberalesMusaceae

N.W.Simmonds, Kew Bull. 11, 3: 463–489. 1956

93F6E37F-9A68-5A51-8482-6F52EF867553

##### Type.

Trinidad, ICTA introduction no. 403, Simmonds 18834 (holo: K!).

#### Musa acuminata Colla subsp. siamea

Taxon classificationPlantaeZingiberalesMusaceae

N.W.Simmonds f. byssina Swangpol & Athawongsa f. nov.

6F5F96CC-760F-5A93-8ECC-08D3AD8501B1

##### Diagnosis.

*Musa acuminata* subsp. *siamea* f. *byssina* is distinguished from other forma by its yellow bracts, whereas bracts that are purple, bluish-purple, or purple-brown with yellow tips are commonly found in f. *siamea* and f. *nanakornii*.

##### Type.

**Thailand** • Pak Chong District, Nakhon Ratchasima Province, 20 January 2006, with male-phase inflorescence, Swangpol & Samana 173 (holotype: BKF!); • Pong Nam Ron District, Chanthaburi Province, 21 January 2006, with male-phase inflorescence, Swangpol & Samana 177 (paratype: BKF!)

##### Description.

***Pseudostem*** of main shoot 2–3 m tall, 30–40 cm in circumference, light green, greenish yellow to medium green, with 3–4 suckers. ***Leaf*** petiole 45–60 cm long, with or without dark purple brown blotches, petiolar canal with erect margins; leaf blade 170–200 cm by 35–60 cm, covered by thin to thick layer of wax; basally oblique, left side cuneate, right side rounded, to cuneate. ***Inflorescence*** peduncle 20–40 cm long, with dense cover of short hairs, in the female phase fusiform to conical in outline, 20–40 cm by 10–15 cm; in the male phase 15–25 cm by 5–10 cm, with apices of the inner rachis bracts exposed, on the both side yellow, covered by thin layer of wax; bracts revolute before falling. ***Rachis*** drooping at male anthesis, rachis diameter usually less than 2.5 cm in diameter, green. ***Flowers*** with ivory-colored perianth and yellow petals lobes; female flowers with an ovate, inner adaxial tepal, green ovary, and ivory-colored stigma; male flowers with an elliptic free adaxial inner tepal, ivory-colored ovary, and yellow to orange stigma. Infructescence. ***Infructescence*** drooping, with 6–10 hands per bunch and 10–25 fruits per hand. ***Fruit*** pedicel 0.5–1.6 cm long; berry 7–10 cm by 1.8–2 cm; apex bottle-necked, 0.5–1.0 cm. ***Seeds*** brown to black, angular in cross section, with rough surface.

##### Distribution and habitat.

Endemic to Thailand. Found rarely in Chanthaburi, Nakhon Ratchasima, and Kamphaeng Phet provinces.

##### Ecology.

Grows in shade at the edge of the forest and in disturbed areas, on low slopes or in open areas.

##### Etymology.

The epithet *byssina* refers to the yellow color of a raw silk cocoon, representing the color of the plant’s inflorescence bracts.

##### Vernacular.

Kluai Pa Pli Leung (“yellow bud banana”—in Thai), Suwannakhattali (“golden banana”—in Thai), and Kluai Pa Mai Thong (“golden silk banana”—in Thai).

##### Notes.

A yellow mutant occurring naturally but rarely. The degree of yellowish-pink coloration varies among specimens found in Kamphaeng Phet, Nakhon Ratchasima, and Chanthaburi, and they are suspected to be unrelated mutants.

#### Musa acuminata Colla subsp. siamea

Taxon classificationPlantaeZingiberalesMusaceae

N.W.Simmonds f. nanakornii Swangpol, Chom. & Sukkaew. f. nov.

E6B55A5F-4E9F-5E56-A159-9F0A1C186C78

Fig. 10

##### Diagnosis.

*Musa acuminata* subsp. *siamea* f. *nanakornii* is similar to f. *siamea* based on its oblique to cuneate leaf base. It is distinguished from f. *siamea* by male inflorescences per rachis numerous, each with long rachilla branches from each flower bud. Female flowers, mostly infertile, occasionally produce fruits with a few seeds.

##### Type.

**Thailand** • Khek Noi, Phetchabun Province, 2 February 2006, with male-phase inflorescence, Sukkaewmanee s.n. (holotype: BKF!); • Phop Phra District, Tak Province, 4 October 2010, with male-phase inflorescence, Swangpol & Somana 442 (paratype: BKF!)

##### Description.

***Pseudostem*** of main shoot 2–3 m tall, 40–50 cm in circumference, light green to green yellow, with 2–6 suckers. ***Leaf*** petiole 70–80 cm long, with or without brown blotches, petiolar canal with erect margins; leaf blade 200–250 cm by 60–80 cm, covered by thin to thick layer of wax; basally oblique. ***Inflorescence*** peduncle 40–45 cm long, with dense cover of short hairs, in the female phase fusiform to ovate, 30–40 cm by 25–30 cm, female flowers mostly infertile; in the male phase inflorescences per rachis numerous, rachis bracts of both sides purple, purple-brown, red-purple to pink-purple, yellow apices, on the inner (adaxial) side covered by thin layer of wax; bracts revolute before falling. ***Rachis*** drooping, with rachilla branches of lateral buds and abnormal flowers. ***Infructescence*** drooping, with 4–6 hands per bunch and 8–20 fruits per hand. ***Fruit*** is often undeveloped.

**Figure 10.**
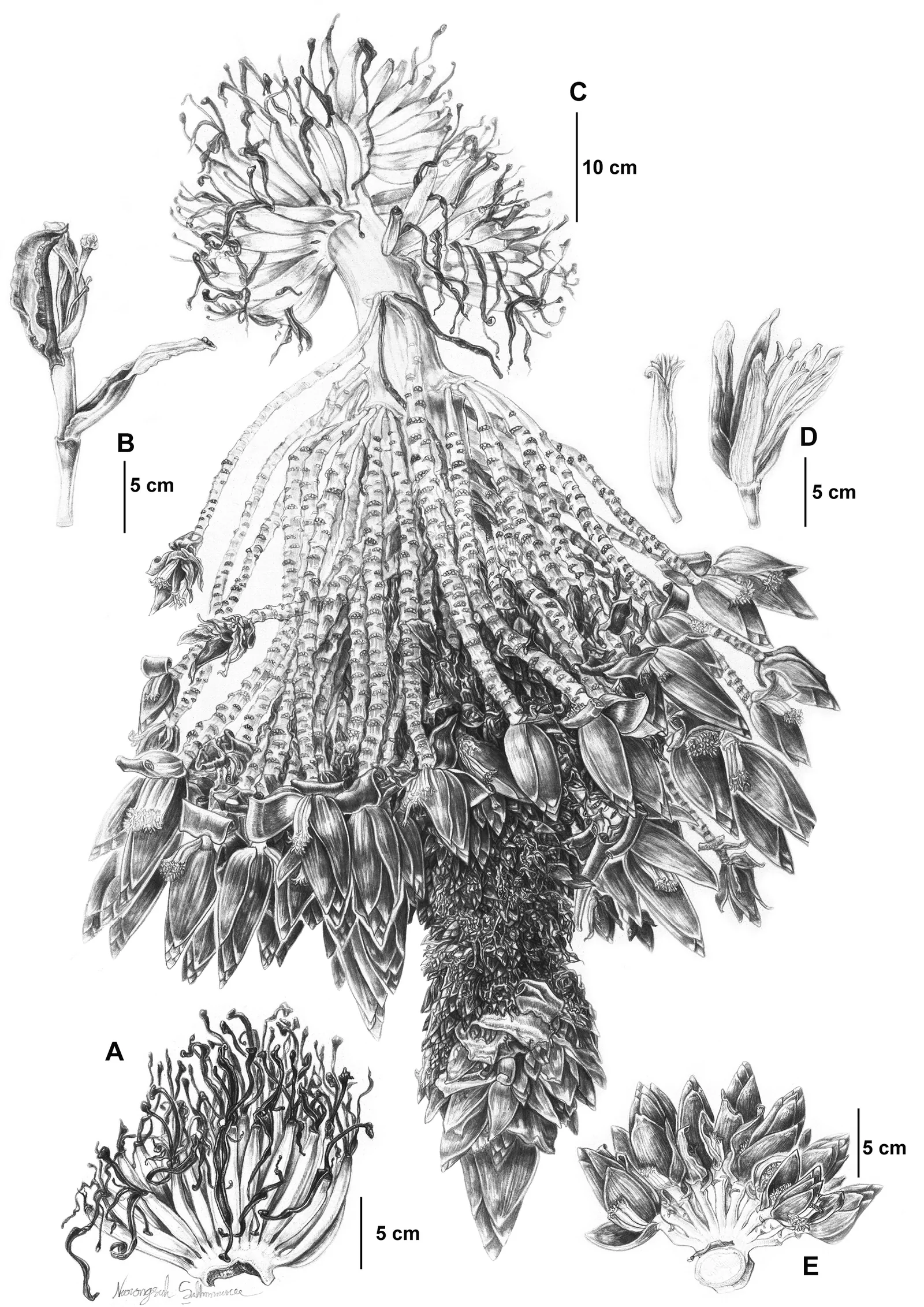
*Musa acuminata* subsp. *siamea* f. *nanakornii* Swangpol, Chom. & Sukkaew, f. nov. **A**. A hand of withering female flowers; **B**. An aberrant female flower; **C**. An inflorescence with hands of aborted female flowers and a primary male-phase inflorescence with numerous hands of secondary male-phase inflorescences on extended rachillas; **D**. Two male flowers, left, a normal flower, and right, an abnormal flower; **E**. A hand of secondary male-phase inflorescences without an extended rachilla. Illustrated by Narongsak Sukkaewmanee; graphic by Sasivimon Swangpol and Wandee Inta.

**Figure 11.**
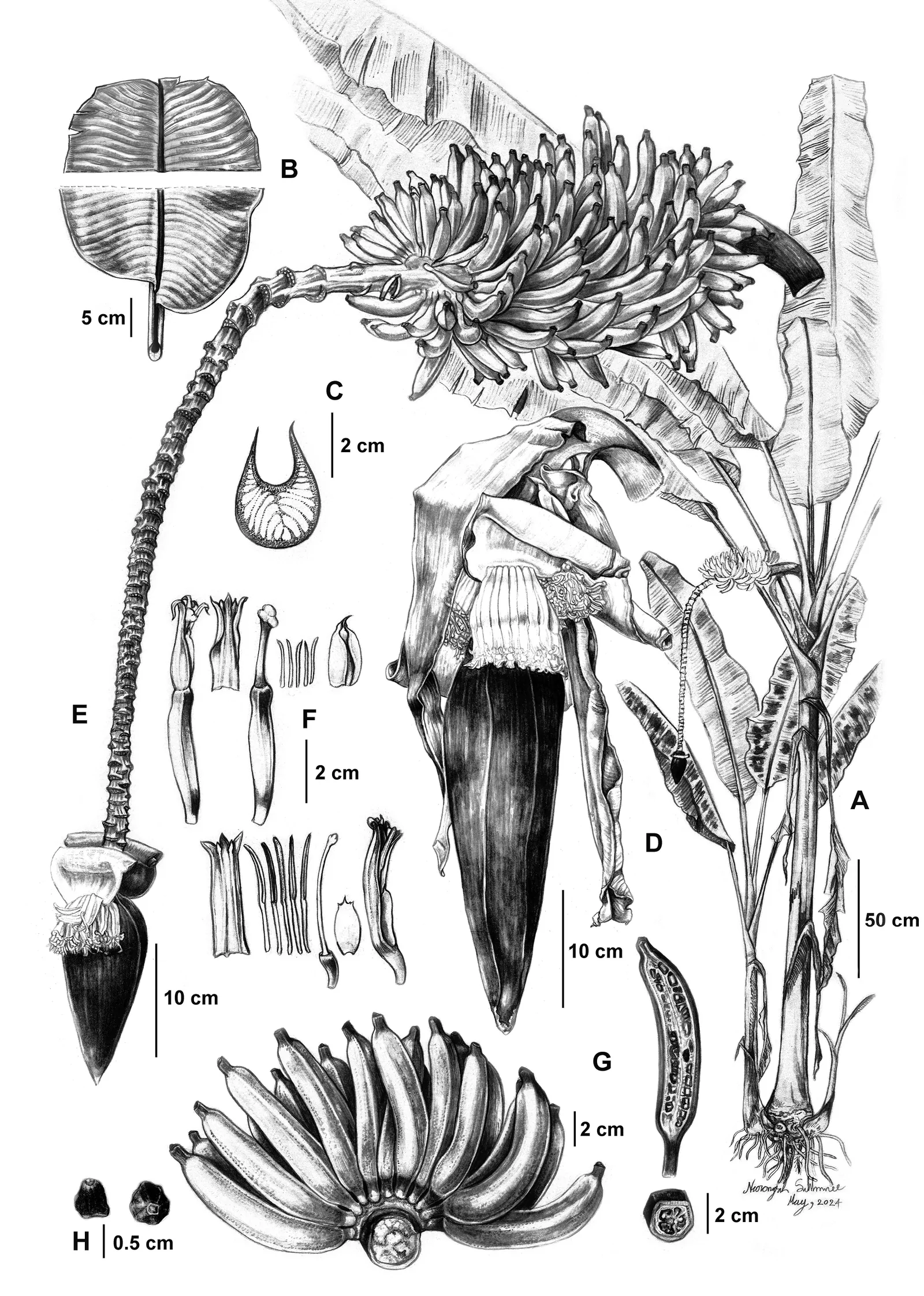
*Musa acuminata* subsp. truncata (Ridl.) Kiew. **A**. Clump; **B**. A leaf showing the base and apex; **C**. A transverse section of the petiole; **D**. A female-phase inflorescence; **E**. A male-phase inflorescence; **F**. Flowers showing, upper row from left to right, a female flower, a compound tepal, a pistil, five anthers, and a free tepal of a female flower, and lower row from left to right, a compound tepal, five anthers, a pistil, a free tepal of a male flower, and a male flower; **G**. Left, a hand of fruits, and right, transverse and longitudinal sections of fruits. Drawn by Narongsak Sukkaewmanee; graphic by Sasivimon C. Swangpol and Wandee Inta.

##### Distribution and habitat.

In cultivation in northern and northeastern Thailand (Tak, Phetchabun, and Nan). Probably also in Laos.

##### Etymology.

The formal name is dedicated to Dr. Weerachai Nanakorn, former President of the Botanical Society under the Patronage of HM Queen Sirikit of Thailand and former Director of the Queen Sirikit Botanic Garden (QSBG), who introduced this clone into cultivation at QSBG in 2009.

##### Vernacular.

Kluai Roi Pli (“one-hundred-bud banana”—in Thai), Kluai Khom Raya (“chandelier banana”—in Thai).

##### Notes.

The banana is a natural mutant. Its numerous male-phase inflorescences are differentiated from the original male flowers and have extended rachillas of varying lengths. All accessions found in three provinces were grown by Hmong hill-tribe villagers as ornamental plants. The male-phase inflorescences are sometimes eaten because they are believed to promote fertility in both men and women.

#### Musa acuminata Colla subsp. truncata

Taxon classificationPlantaeZingiberalesMusaceae

(Ridl.) Kiew, Ann. Bot. 88, 6: 1025. 2001

E57D17D1-C435-50EC-89F1-FEA59AA8FAFD

##### Type.

Malaysia [Selangor, Pahang], 1905, Henry Ridley 13694 (lectotype: SING).

##### Description.

***Pseudostem*** of main shoot 3–6 m tall, 30–45 cm in circumference, light green, with 2–4 suckers, often with large blotches. ***Leaf*** petiole 50–100 cm long, with small to large, dark purple-brown blotches, petiolar margins erect; leaf blade 155–245 cm by 55–75 cm, with little or no visible sign of wax; base obliquely rounded, typically with a more distal left-handed side. ***Inflorescence*** peduncle 18–43 cm long, with short hairs varying in density, in the female phase fusiform to conical in outline, 20–40 cm by 10–15 cm, in the male phase with ovate rachis bracts ovate, 10–23 cm by 7–10 cm; apices of the inner rachis bracts completely concealed by the outer bracts, apically acute, revolute before shedding, ivory-colored or pale red-purple fading to ivory at the base on the inner (adaxial) side and dark purple without yellow tips on the outer (abaxial) side, not persistent after anthesis. ***Rachis*** drooping at male anthesis, rachis diameter usually less than 2.5 cm in diameter, purplish brown to dark purple. ***Flowers*** with ivory-colored perianth and yellow petals lobes; female flowers with an ovate, inner adaxial tepal, green ovary, and ivory-colored stigma; male flowers with an elliptic free adaxial inner tepal, ivory-colored ovary, and yellow to orange stigma. Infructescence. ***Infructescence*** horizontal or slightly curved downward, with 4–15 hands per bunch and 15–28 fruits per hand. ***Fruit*** pedicel 1.2–2 cm long; berry 10–12 cm by 1.5–2 cm. ***Seeds*** brown to black, angular in cross section, with rough surface.

##### Distribution and habitat.

**Thailand** • Peninsular: Narathiwat (Sukhirin, close to Hala-Bala Wildlife Sanctuary Wang, Ban Sai Borisat, Namtok Chat Warin Sungai Padi, along highway no. 4077 Sri Sakorn), Yala (Betong, KERR 7643, BM), Songkla (Ban Pian, KERR 14821 BM).

##### Ecology.

Hill and montane tropical forests, at 90–750 m altitude.

##### Etymology.

The epithet *truncata* refers to the “truncate apex” or square end of the leaves (Ridley 1909).

##### Vernacular.

Kluai Thuean (meaning “wild banana”) was used in southernmost Thailand as mentioned by Simmonds (1956) for *M. acuminata* subsp. *microcarpa* and referred to Kerr specimens in which this vernacular name was applied to both *M. acuminata* subsp. *siamea* and *M. acuminata* subsp. *microcarpa*; Kluai Pa (also means “wild banana”), Kluai Pa Pli Muang (“purple-bud wild banana”—in Thai).

##### Notes.

A new record for Thailand. Simmonds (1956) previously mentioned this subspecies native to Thailand as *M. acuminata* subsp. *microcarpa*. Hybrids of *M. acuminata* subsp. *truncata* and *M. acuminata* subsp. *malaccensis* were found growing together in Changwat Yala. The hybrids possess bracts with an intense red color internally.

## Supplementary Material

XML Treatment for M. acuminata

XML Treatment for Musa acuminata Colla subsp. kraburiensis

XML Treatment for M. acuminata Colla subsp. kraburiensis

XML Treatment for Musa acuminata Colla subsp. malaccensis

XML Treatment for Musa acuminata Colla subsp. siamea

XML Treatment for Musa acuminata Colla subsp. siamea

XML Treatment for Musa acuminata Colla subsp. siamea

XML Treatment for Musa acuminata Colla subsp. truncata

## Additional information

### Artificial Intelligence (AI) use

The authors accept full responsibility for the content of the manuscript, including the disclosure of any use of AI.

Figures 2, 4 were digitally sharpened and upscaled to 600-1000 DPI using standard image-processing methods (noise reduction, unsharp masking, Lanczos interpolation); no AI-generated content was added and no morphological details were altered.

### Author ORCIDs

W. Inta https://orcid.org/0009-0003-7409-7052

S.C. Swangpol https://orcid.org/0000-0002-0317-4546

K. Athawongsa https://orcid.org/0009-0006-0748-0256

S. Wongniam https://orcid.org/0009-0006-2109-2022

N. Sukkaewmanee https://orcid.org/0009-0008-4999-7733

T. Seelanan https://orcid.org/0000-0002-7768-6130

P. Srikrainoon https://orcid.org/0009-0001-8915-7789

W. Nilapaka https://orcid.org/0009-0006-9311-4884

T. Rotchanapreeda https://orcid.org/0009-0009-5683-7225

J. Somana https://orcid.org/0000-0002-2883-0693

### Data availability

All of the data that support the findings of this study are available in the main text or Supplementary Information.

## Supplementary materials

Supplementary material 1 A List of 221 banana accessions including 211 *Musa acuminata* and 10 accessions from two outgroup species investigated in this study Data type docx Explanation note Among the *M. acuminata* specimens, 204 accessions were used for the location map in Fig. 1 (Column Analyses ‘L’ in this table); 195 accessions were used for UPGMA analysis based on morphological characters (Column Analyses ‘M’) and 20 accessions were used for phylogenetic analysis (Column Analyses ‘P’). Meanwhile, 199 (Column Analyses ‘A’) were used for the altitude analysis in Fig. 2. https://binary.pensoft.net/file/1745898This dataset is made available under the Open Database License (http://opendatacommons.org/licenses/odbl/1.0/). The Open Database License (ODbL) is a license agreement intended to allow users to freely share, modify, and use this Dataset while maintaining this same freedom for others, provided that the original source and author(s) are credited. Wandee Inta, Sasivimon Chomchalow Swangpol

Supplementary material 2 Data matrix of morphological characters used in multivariate analysis Data type docx Explanation note Codings appeared based on those in Table 2. From all four subspecies found in Thailand, 195 *Musa* accessions were investigated. https://binary.pensoft.net/file/1745899This dataset is made available under the Open Database License (http://opendatacommons.org/licenses/odbl/1.0/). The Open Database License (ODbL) is a license agreement intended to allow users to freely share, modify, and use this Dataset while maintaining this same freedom for others, provided that the original source and author(s) are credited.Author: Wandee Inta
