# Supplementary material for: Delimitation of the wild banana Musa acuminata subspecific complex (Musaceae) in Thailand
Source: PhytoKeys. 2026 Aug 14;278:127–56. doi: 10.3897/phytokeys.278.165557 (PMC13498946; doi:10.3897/phytokeys.278.165557)
Supplement: Supplementary file 1 [file phytokeys-278-127_article-165557__-s001.docx]

**Table 1S (Supplement)** A List of 221 banana accessions including 211 *Musa acuminata* and 10 accessions from two outgroup species investigated in this study. Among the *M. acuminata* specimens, 204 accessions were used for the location map in Figure 1 (Column Analyses ‘L’ in this table) and 195 accessions were used for UPGMA analysis based on morphological characters (Column Analyses ‘M’). Meanwhile, 199 (Column Analyses ‘A’) were used for the altitude analysis in Fig. 2.

| No. | Scientific name | Forma | Collection site District | Collection site Province | Accession Number | Sample code | Analyses | | |
| --- | --- | --- | --- | --- | --- | --- | --- | --- | --- |
|  |  |  |  |  |  |  | L | A | M |
| 1 | *M. acuminata* subsp. *kraburiensis* | *kraburiensis* | Kra Buri | Narathiwat | SS & JS 009 | AK009 | - | A | M |
| 2 | *M. acuminata* subsp*. kraburiensis* | *kraburiensis* | Mueang | Chumphon | SS & JS 104 | AK104 | L | A | M |
| 3 | *M. acuminata* subsp. *kraburiensis* | *kraburiensis* | Lang Suan | Chumphon | SS & JS 105 | AK105 | L | A | M |
| 4 | *M. acuminata* subsp. *kraburiensis* | *kraburiensis* | Mueang | Chumphon | SS & JS 106 | AK106 | L | A | M |
| 5 | *M. acuminata* subsp. *kraburiensis* | *kraburiensis* | Pha To | Chumphon | SS & JS 107 | AK107 | L | A | M |
| 6 | *M. acuminata* subsp*.* *kraburiensis* | *kraburiensis* | Suk Samran | Ranong | SS & JS 108 | AK108 | L | A | M |
| 7 | *M. acuminata* subsp*. kraburiensis* | *kraburiensis* | Lang Suan | Chumphon | SS & JS 118 | AK118 | - | A | M |
| 8 | *M. acuminata* subsp*. kraburiensis* | *kraburiensis* | Kra Buri | Ranong | SS & JS 137 | AK137 | L | A | M |
| 9 | *M. acuminata* subsp*. kraburiensis* | *kraburiensis* | Suk Samran | Ranong | SS & JS 142 | AK142 | L | A | M |
| 10 | *M. acuminata* subsp*. kraburiensis* | *kraburiensis* | Mueang | Ranong | SS & JS 144 | AK144 | - | A | M |
| 11 | *M. acuminata* subsp*. kraburiensis* | *kraburiensis* | Sawi | Chumphon | SS & JS 145 | AK145 | L | A | M |
| 12 | *M. acuminata* subsp*. kraburiensis* | *kraburiensis* | Tha Sae | Chumphon | SS & JS 146 | AK146 | L | A | M |
| 13 | *M. acuminata* subsp*. kraburiensis* | *kraburiensis* | Sawi | Chumphon | SS & JS 374 | AK374 | L | A | M |
| 14 | *M. acuminata* subsp*. kraburiensis* | *kraburiensis* | Sawi | Chumphon | SS & JS 375 | AK375 | L | A | M |
| 15 | *M. acuminata* subsp*. kraburiensis* | *kraburiensis* | Tha Sae | Chumphon | SS & JS 381 | AK381 | - | A | M |
| 16 | *M. acuminata* subsp*. kraburiensis* | *kraburiensis* | Tha Sae | Chumphon | SS & JS 382 | AK382 | - | A | M |
| 17 | *M. acuminata* subsp*. kraburiensis* | *kraburiensis* | Lang Suan | Chumphon | SS & JS 598 | AK598 | - | A | M |
| 18 | *M. acuminata* subsp*. kraburiensis* | *kraburiensis* | Lang Suan | Chumphon | SS & JS 599 | AK599 | L | A | M |
| 19 | *M. acuminata* subsp*. kraburiensis* | *kraburiensis* | Tha Sae | Chumpon | SS & JS 665 | AK665 | - | - | - |
| 10 | *M. acuminata* subsp*. kraburiensis* | *kraburiensis* | Pathio | Chumphon | SS & JS 685 | AK685 | L | A | M |
| 21 | *M. acuminata* subsp*. kraburiensis* | *kraburiensis* | Tha Sae | Chumphon | SS & JS 686 | AK686 | - | A | - |
| 22 | *M. acuminata* subsp*. kraburiensis* | *kraburiensis* | Tha Sae | Chumphon | SS & JS 688 | AK688 | L | A | M |
| 23 | *M. acuminata* subsp*. kraburiensis* | *kraburiensis* | Kapoe | Ranong | SS & JS 689 | AK689 | L | A | M |
| 24 | *M. acuminata* subsp*. kraburiensis* | *kraburiensis* | Kapoe | Ranong | SS & JS 690 | AK690 | L | A | M |
| 25 | *M. acuminata* subsp*. kraburiensis* | *kraburiensis* | Mueang | Chumphon | SS & JS 251 | AK251 | L | A | M |
| 26 | *M. acuminata* subsp*. kraburiensis* | *luteolus* | Mueang | Ranong | SS & JS 600 | AK600 | - | A | M |
| 27 | *M. acuminata* subsp*. kraburiensis* | *luteolus* | Mueang | Ranong | SS & JS 601 | AK601 | - | A | M |
| 28 | *M. acuminata* subsp*. malaccensis* | *malaccensis* | Takua Pa | Phang Nga | SS & JS 005 | AM005 | - | A | M |
| 29 | *M. acuminata* subsp*. malaccensis* | *malaccensis* | Takua Pa | Phang Nga | SS & JS 006 | AM006 | - | A | M |
| 30 | *M. acuminata* subsp*. malaccensis* | *malaccensis* | Khura Buri | Phang Nga | SS & JS 010 | AM010 | - | A | M |
| 31 | *M. acuminata* subsp*. malaccensis* | *malaccensis* | Mueang | Krabi | SS & JS 109 | AM109 | - | A | M |
| 32 | *M. acuminata* subsp*. malaccensis* | *malaccensis* | Mueang | Krabi | SS & JS 111 | AM111 | - | A | M |
| 33 | *M. acuminata* subsp*. malaccensis* | *malaccensis* | Ao Luek | Krabi | SS & JS 112 | AM112 | - | A | M |
| 34 | *M. acuminata* subsp*. malaccensis* | *malaccensis* | Chang Klang | Nakhon Si Thammarat | SS & JS 113 | AM113 | - | A | M |
| 35 | *M. acuminata* subsp*. malaccensis* | *malaccensis* | Phrom Khiri | Nakhon Si Thammarat | SS & JS 114 | AM114 | - | A | M |
| 36 | *M. acuminata* subsp*. malaccensis* | *malaccensis* | Si Chon | Nakhon Si Thammarat | SS & JS 115 | AM115 | L | A | M |
| 37 | *M. acuminata* subsp*. malaccensis* | *malaccensis* | Kanchanadit | Surat Thani | SS & JS 116 | AM116 | - | A | M |
| 38 | *M. acuminata* subsp*. malaccensis* | *malaccensis* | Takua Pa | Phang Nga | SS & JS 138 | AM138 | - | A | M- |
| 39 | *M. acuminata* subsp*. malaccensis* | *malaccensis* | Mueang | Phang nga | SS & JS 139 | AM139 | L | A | M |
| 40 | *M. acuminata* subsp*. malaccensis* | *malaccensis* | Phanom | Surat Thani | SS & JS 140 | AM140 | L | A | M |
| 41 | *M. acuminata* subsp*. malaccensis* | *malaccensis* | Phanom | Surat Thani | SS & JS 141 | AM141 | - | A | M |
| 42 | *M. acuminata* subsp*. malaccensis* | *malaccensis* | Na Thawi | Songkhla | SS & JS 199 | AM199 | - | A | M |
| 43 | *M. acuminata* subsp*. malaccensis* | *malaccensis* | Na Thawi | Songkhla | SS & JS 200 | AM200 | - | A | M |
| 44 | *M. acuminata* subsp*. malaccensis* | *malaccensis* | Bannangsata | Yala | SS & JS 202 | AM202 | - | A | M |
| 45 | *M. acuminata* subsp*. malaccensis* | *malaccensis* | Bannangsata | Yala | SS & JS 203 | AM203 | - | A | M |
| 46 | *M. acuminata* subsp*. malaccensis* | *malaccensis* | Than To | Yala | SS & JS 204 | AM204 | - | A | M |
| 47 | *M. acuminata* subsp*. malaccensis* | *malaccensis* | Betong | Yala | SS & JS 205 | AM205 | L | A | M |
| 48 | *M. acuminata* subsp*. malaccensis* | *malaccensis* | Hat Yai | Songkhla | SS & JS 209 | AM209 | - | A | M |
| 49 | *M. acuminata* subsp*. malaccensis* | *malaccensis* | Khuan Don | Satun | SS & JS 210 | AM210 | L | A | M |
| 50 | *M. acuminata* subsp*. malaccensis* | *malaccensis* | Thung Wa | Satun | SS & JS 211 | AM211 | - | A | M |
| 51 | *M. acuminata* subsp*. malaccensis* | *malaccensis* | Thung Wa | Satun | SS & JS 212 | AM212 | - | A | M |
| 52 | *M. acuminata* subsp*. malaccensis* | *malaccensis* | Yan Ta Khao | Trang | SS & JS 213 | AM213 | - | A | M |
| 53 | *M. acuminata* subsp*. malaccensis* | *malaccensis* | Na Yong | Trang | SS & JS 214 | AM214 | L | A | M |
| 54 | *M. acuminata* subsp*. malaccensis* | *malaccensis* | Sri Banphot | Phatthalung | SS & JS 216 | AM216 | L | A | M |
| 55 | *M. acuminata* subsp*. malaccensis* | *malaccensis* | Si Banphot | Phatthalung | SS & JS 217 | AM217 | - | A | M |
| 56 | *M. acuminata* subsp*. malaccensis* | *malaccensis* | Nopphitam | Narathiwat | SS & JS 218 | AM218 | - | A | M |
| 57 | *M. acuminata* subsp*. malaccensis* | *malaccensis* | Chulabhorn | Nakhon Si Thammarat | SS & JS 219 | AM219 | - | A | M |
| 58 | *M. acuminata* subsp*. malaccensis* | *malaccensis* | Khao Chai Son | Phatthalung | SS & JS 220 | AM220 | - | A | M |
| 59 | *M. acuminata* subsp*. malaccensis* | *malaccensis* | Mae Sot | Tak | SS & JS 228 | AM228 | - | A | M |
| 60 | *M. acuminata* subsp*. malaccensis* | *malaccensis* | Mae Sod | Tak | SS & JS 274 | AM274 | L | A | M |
| 61 | *M. acuminata* subsp*. malaccensis* | *malaccensis* | Sop Moei | Mae Hong Son | SS & JS 281 | AM281 | - | A | M |
| 62 | *M. acuminata* subsp*. malaccensis* | *malaccensis* | Phop Phra | Tak | SS & JS 309 | AM309 | - | A | M |
| 63 | *M. acuminata* subsp*. malaccensis* | *malaccensis* | Mae Sariang | Mae Hong Son | SS & JS 357 | AM357 | - | A | M |
| 64 | *M. acuminata* subsp*. malaccensis* | *malaccensis* | Thalang | Phuket | SS & JS 376 | AM376 | L | A | M |
| 65 | *M. acuminata* subsp*. malaccensis* | *malaccensis* | Wang Wiset | Trang | SS & JS 377 | AM377 | L | A | M |
| 66 | *M. acuminata* subsp*. malaccensis* | *malaccensis* | Thung Wa | Satun | SS & JS 378 | AM378 | L | A | M |
| 67 | *M. acuminata* subsp*. malaccensis* | *malaccensis* | Had Yai | Songkhla | SS & JS 379 | AM379 | L | A | M |
| 68 | *M. acuminata* subsp*. malaccensis* | *malaccensis* | Tha Chana | Narathiwat | SS & JS 380 | AM380 | - | A | M |
| 69 | *M. acuminata* subsp*. malaccensis* | *malaccensis* | Tha Song Yang | Tak | SS & JS 428 | AM428 | - | A | - |
| 70 | *M. acuminata* subsp*. malaccensis* | *malaccensis* | Phop Phra | Tak | SS & JS 445 | AM445 | - | A | M |
| 71 | *M. acuminata* subsp*. malaccensis* | *malaccensis* | Khok Pho | Pattani | SS & JS 477 | AM477 | - | A | M |
| 72 | *M. acuminata* subsp*. malaccensis* | *malaccensis* | Khok Pho | Pattani | SS & JS 478 | AM478 | - | A | M |
| 73 | *M. acuminata* subsp*. malaccensis* | *malaccensis* | Khok Pho | Pattani | SS & JS 479 | AM479 | - | A | - |
| 74 | *M. acuminata* subsp*. malaccensis* | *malaccensis* | Bacho | Narathiwat | SS & JS 480 | AM480 | - | A | - |
| 75 | *M. acuminata* subsp*. malaccensis* | *malaccensis* | Sukhirin | Narathiwat | SS & JS 482 | AM482 | - | A | M |
| 76 | *M. acuminata* subsp*. malaccensis* | *malaccensis* | Waeng | Narathiwat | SS & JS 485 | AM485 | - | A | M |
| 77 | *M. acuminata* subsp*. malaccensis* | *malaccensis* | Sukhirin | Narathiwat | SS & JS 489 | AM489 | - | A | M |
| 78 | *M. acuminata* subsp*. malaccensis* | *malaccensis* | Waeng | Narathiwat | SS & JS 492 | AM492 | - | A | - |
| 79 | *M. acuminata* subsp*. malaccensis* | *malaccensis* | Ra-ngae | Narathiwat | SS & JS 494 | AM494 | - | A | - |
| 80 | *M. acuminata* subsp*. malaccensis* | *malaccensis* | Than To | Yala | SS & JS 496 | AM496 | - | A | M |
| 81 | *M. acuminata* subsp*. malaccensis* | *malaccensis* | Mae La Noi | Mae Hong Son | SS & JS 546 | AM546 | - | A | M |
| 82 | *M. acuminata* subsp*. malaccensis* | *malaccensis* | Tha Sae | Chomphon | SS & JS 584 | AM584 | - | A | - |
| 83 | *M. acuminata* subsp*. malaccensis* | *malaccensis* | Kra Buri | Ranong | SS & JS 602 | AM602 | L | A | M |
| 84 | *M. acuminata* subsp. *malaccensis* | *malaccensis* | Tha Sae | Chomphon | SS & JS 687 | AM687 | L | A | - |
| 85 | *M. acuminata* subsp*. malaccensis* | *malaccensis* | Takua Pa | Phang Nga | SS & JS 691 | AM691 | L | A | M |
| 86 | *M. acuminata* subsp*. malaccensis* | *malaccensis* | Takua Pa | Phang Nga | SS & JS 692 | AM692 | L | A | M |
| 87 | *M. acuminata* subsp*. malaccensis* | *malaccensis* | Si Sakhon | Narathiwat | SS & JS 699 | AM699 | L | A | M |
| 88 | *M. acuminata* subsp*. siamea* | *byssinus* | Pak Chong | Nakhon Ratchasima | SS & JS 173 | AS173 | L | A | M |
| 89 | *M. acuminata* subsp*. siamea* | *byssinus* | Pong Nam Ron | Chanthaburi | SS & JS 177 | AS177 | L | A | M |
| 90 | *M. acuminata* subsp*. siamea* | *byssinus* | Wang Nam Khieo | Nakhon Ratchasima | SS & JS 224 | AS224 | L | - | M |
| 91 | *M. acuminata* subsp*. siamea* | *byssinus* | Sila Thong | Kamphaeng Phet | SS & JS 307 | AS307 | L | - | M |
| 92 | *M. acuminata* subsp*. siamea* | *byssinus* |  | Uthai Thani | SS & JS 670 | AS670 | L | - | _ |
| 93 | *M. acuminata* subsp*. siamea* | *nanakornii* | Khao Kho | Phetchabun | SS & JS 338 | AS338 | L | A | M |
| 94 | *M. acuminata* subsp*. siamea* | *nanakornii* | Phob Phra | Tak | SS & JS 442 | AS442 | - | A | M |
| 95 | *M. acuminata* subsp*. siamea* | *nanakornii* | Khao Kho | Phetchabun | SS & JS 405 | AS405 | - | A | - |
| 96 | *M. acuminata* subsp*. siamea* | *nanakornii* | Chatuchak | Bangkok | SS & JS 704 | AS704 | - | A | - |
| 97 | *M. acuminata* subsp*. siamea* | *siamea* | Khao Kho | Phetchabun | SS & JS 001 | AS001 | - | A | M |
| 98 | *M. acuminata* subsp*. siamea* | *siamea* | Pak Chong | Nakhon Ratchasima | SS & JS 007 | AS007 | L | A | M |
| 99 | *M. acuminata* subsp*. siamea* | *siamea* | Thong Pha Phum | Kanchanaburi | SS & JS 008 | AS008 | - | A | M |
| 100 | *M. acuminata* subsp*. siamea* | *siamea* | Den Chai | Prae | SS & JS 019 | AS019 | L | A | M |
| 101 | *M. acuminata* subsp*. siamea* | *siamea* | Mueang | Uttaradit | SS & JS 021 | AS021 | L | A | M |
| 102 | *M. acuminata* subsp*. siamea* | *siamea* | Khon San | Chaiyaphum | SS & JS 089 | AS089 | - | A | M |
| 103 | *M. acuminata* subsp*. siamea* | *siamea* | Nam Nao | Phetchabun | SS & JS 090 | AS090 | - | A | M |
| 104 | *M. acuminata* subsp*. siamea* | *siamea* | Nam Nao | Phetchabun | SS & JS 091 | AS091 | - | A | M |
| 105 | *M. acuminata* subsp*. siamea* | *siamea* | Nam Nao | Phetchabun | SS & JS 092 | AS092 | - | A | M |
| 106 | *M. acuminata* subsp*. siamea* | *siamea* | Pak Chom | Loei | SS & JS 094 | AS094 | - | A | M |
| 107 | *M. acuminata* subsp*. siamea* | *siamea* | Chiang Khan | Loei | SS & JS 096 | AS096 | - | A | M |
| 108 | *M. acuminata* subsp*. siamea* | *siamea* | Dan Sai | Loei | SS & JS 097 | AS097 | - | A | M |
| 109 | *M. acuminata* subsp*. siamea* | *siamea* | Nakhon Thai | Phitsanulok | SS & JS 098 | AS098 | - | A | M |
| 110 | *M. acuminata* subsp*. siamea* | *siamea* | Wang Thong | Phitsanulok | SS & JS 099 | AS099 | - | A | M |
| 111 | *M. acuminata* subsp*. siamea* | *siamea* | Khao Kho | Phetchabun | SS & JS 101 | AS101 | - | A | M |
| 112 | *M. acuminata* subsp*. siamea* | *siamea* | Phutthachai | Saraburi | SS & JS 102 | AS102 | - | A | M |
| 113 | *M. acuminata* subsp*. siamea* | *siamea* | Pang Mapha | Mae Hong Son | SS & JS 126 | AS126 | - | A | M |
| 114 | *M. acuminata* subsp*. siamea* | *siamea* | Khlong Lan | Kamphaeng Phet | SS & JS 129 | AS129 | - | A | M |
| 115 | *M. acuminata* subsp*. siamea* | *siamea* | Hua Hin | Prachuap Khiri Khan | SS & JS 131 | AS131 | - | A | M |
| 116 | *M. acuminata* subsp*. siamea* | *siamea* | Pak Chong | Nakhon Ratchasima | SS & JS 132 | AS132 | - | A | M |
| 117 | *M. acuminata* subsp*. siamea* | *siamea* | Bang Saphan | Prachuap Khiri Khan | SS & JS 136 | AS136 | - | - | - |
| 118 | *M. acuminata* subsp*. siamea* | *siamea* | Mueang | Prachuap Khiri Khan | SS & JS 147 | AS147 | L | A | M |
| 119 | *M. acuminata* subsp*. siamea* | *siamea* | Pak Chong | Nakhon Ratchasima | SS & JS 172 | AS172 | - | A | M |
| 120 | *M. acuminata* subsp*. siamea* | *siamea* | Mueang | Sa Kaeo | SS & JS 174 | AS174 | - | A | M |
| 121 | *M. acuminata* subsp*. siamea* | *siamea* | Soi Dao | Chanthaburi | SS & JS 176 | AS176 | - | A | M |
| 122 | *M. acuminata* subsp*. siamea* | *siamea* | Mueang | Chanthaburi | SS & JS 180 | AS180 | - | A | M |
| 123 | *M. acuminata* subsp*. siamea* | *siamea* | Khao Khitchakut | Chanthaburi | SS & JS 181 | AS181 | - | A | M |
| 124 | *M. acuminata* subsp*. siamea* | *siamea* | Klaeng | Rayong | SS & JS 182 | AS182 | L | A | M |
| 125 | *M. acuminata* subsp*. siamea* | *siamea* | Laplae | Uttaradit | SS & JS 185 | AS185 | - | A | M |
| 126 | *M. acuminata* subsp*. siamea* | *siamea* | Khlong Lan | Kamphaeng Phet | SS & JS 197 | AS197 | - | A | M |
| 127 | *M. acuminata* subsp*. siamea* | *siamea* | Wang Nam Khieo | Nakhon Ratchasima | SS & JS 221 | AS221 | - | A | M |
| 128 | *M. acuminata* subsp*. siamea* | *siamea* | Mueang | Nakhon Nayok | SS & JS 225 | AS225 | L | A | M |
| 129 | *M. acuminata* subsp*. siamea* | *siamea* | Klang | Rayong | SS & JS 227 | AS227 | - | A | M |
| 130 | *M. acuminata* subsp*. siamea* | *siamea* | Ko Chang | Trat | SS & JS 232 | AS232 | - | A | M |
| 131 | *M. acuminata* subsp*. siamea* | *siamea* | Mae Rim | Chiang Mai | SS & JS 233 | AS233 | - | A | M |
| 132 | *M. acuminata* subsp*. siamea* | *siamea* | Suan Phueng | Ratchaburi | SS & JS 238 | AS238 | - | A | M |
| 133 | *M. acuminata* subsp*. siamea* | *siamea* | Thong Pha Phum | Kanchanaburi | SS & JS 245 | AS245 | - | A | M |
| 134 | *M. acuminata* subsp*. siamea* | *siamea* | Sangkhla Buri | Kanchanaburi | SS & JS 247 | AS247 | - | A | M |
| 135 | *M. acuminata* subsp*. siamea* | *siamea* | Suan Phueng | Ratchaburi | SS & JS 250 | AS250 | - | A | M |
| 136 | *M. acuminata* subsp*. siamea* | *siamea* | Doi Saket | Chiang Mai | SS & JS 258 | AS258 | - | A | M |
| 137 | *M. acuminata* subsp*. siamea* | *siamea* | Wiang Pa Pao | Chiang Mai | SS & JS 262 | AS262 | - | A | M |
| 138 | *M. acuminata* subsp*. siamea* | *siamea* | Chiang Dao | Chiang Mai | SS & JS 264 | AS264 | - | A | M |
| 139 | *M. acuminata* subsp*. siamea* | *siamea* | Hang Chat | Lampang | SS & JS 266 | AS266 | - | A | M |
| 140 | *M. acuminata* subsp*. siamea* | *siamea* | Mueang Pan | Lampang | SS & JS 268 | AS268 | - | A | M |
| 141 | *M. acuminata* subsp*. siamea* | *siamea* | Pang Sila Thong | Kamphaeng Phet | SS & JS 271 | AS271 | - | A | - |
| 142 | *M. acuminata* subsp*. siamea* | *siamea* | Phang | Chiang Mai | SS & JS 283 | AS283 | L | A | M |
| 143 | *M. acuminata* subsp*. siamea* | *siamea* | Mae Pha Luang | Chiang Rai | SS & JS 286 | AS286 | L | A | M |
| 144 | *M. acuminata* subsp*. siamea* | *siamea* | Chiang Muan | Phayao | SS & JS 289 | AS289 | L | A | M |
| 145 | *M. acuminata* subsp*. siamea* | *siamea* | Ban Luang | Nan | SS & JS 290 | AS290 | L | A | M |
| 146 | *M. acuminata* subsp*. siamea* | *siamea* | Santi Suk | Nan | SS & JS 291 | AS291 | L | A | M |
| 147 | *M. acuminata* subsp*. siamea* | *siamea* | Long | Phrae | SS & JS 293 | AS293 | L | A | M |
| 148 | *M. acuminata* subsp*. siamea* | *siamea* | Mueang | Nakhon Nayok | SS & JS 300 | AS300 | L | A | M |
| 149 | *M. acuminata* subsp*. siamea* | *siamea* | Mueang | Nakhon Nayok | SS & JS 301 | AS301 | - | A | M |
| 150 | *M. acuminata* subsp*. siamea* | *siamea* | Mueang | Nakhon Nayok | SS & JS 303 | AS303 | L | A | M |
| 151 | *M. acuminata* subsp*. siamea* | *siamea* | Pang Sila Thong | Kamphaeng Phet | SS & JS 305 | AS305 | - | A | M |
| 152 | *M. acuminata* subsp*. siamea* | *siamea* | Phop Phra | Tak | SS & JS 310 | AS310 | - | A | M |
| 153 | *M. acuminata* subsp*. siamea* | *siamea* | Umphang | Tak | SS & JS 312 | AS312 | - | A | M |
| 154 | *M. acuminata* subsp*. siamea* | *siamea* | Huai Khot | Uthai Thani | SS & JS 320 | AS320 | - | A | M |
| 155 | *M. acuminata* subsp*. siamea* | *siamea* | Ban Rai | Uthai Thani | SS & JS 321 | AS321 | - | A | M |
| 156 | *M. acuminata* subsp*. siamea* | *siamea* | Dan Chang | Suphanburi | SS & JS 322 | AS322 | - | A | M |
| 157 | *M. acuminata* subsp*. siamea* | *siamea* | Kang Krachan | Phetchaburi | SS & JS 325 | AS325 | - | A | M |
| 158 | *M. acuminata* subsp*. siamea* | *siamea* | Kang Krachan | Phetchaburi | SS & JS 326 | AS326 | - | A | M |
| 159 | *M. acuminata* subsp*. siamea* | *siamea* | Phuphan | Sakon Nakhon | SS & JS 330 | AS330 | - | - | M |
| 160 | *M. acuminata* subsp*. siamea* | *siamea* | Ban Phan | Nakhon Phanom | SS & JS 334 | AS334 | L | A | M |
| 161 | *M. acuminata* subsp*. siamea* | *siamea* | Khao Kho | Phetchabun | SS & JS 339 | AS339 | - | A | M |
| 162 | *M. acuminata* subsp*. siamea* | *siamea* | Chat Trakan | Phitsanulok | SS & JS 340 | AS340 | L | A | - |
| 163 | *M. acuminata* subsp*. siamea* | *siamea* | Wiang Sa | Nan | SS & JS 343 | AS343 | L | A | M |
| 164 | *M. acuminata* subsp*. siamea* | *siamea* | Thoen | Lampang | SS & JS 359 | AS359 | - | A | - |
| 165 | *M. acuminata* subsp*. siamea* | *siamea* | Hua Hin | Prachuap Khiri Khan | SS & JS 372 | AS372 | - | A | M |
| 166 | *M. acuminata* subsp*. siamea* | *siamea* | Hua Hin | Prachuap Khiri Khan | SS & JS 373 | AS373 | - | A | M |
| 167 | *M. acuminata* subsp*. siamea* | *siamea* | Sangkhla Buri | Kanchanaburi | SS & JS 390 | AS390 | - | A | M |
| 168 | *M. acuminata* subsp*. siamea* | *siamea* | Sangkhla Buri | Kanchanaburi | SS & JS 392 | AS392 | - | A | M |
| 169 | *M. acuminata* subsp*. siamea* | *siamea* | Sangkhla Buri | Kanchanaburi | SS & JS 394 | AS394 | - | A | M |
| 170 | *M. acuminata* subsp*. siamea* | *siamea* | Pong | Phayao | SS & JS 400 | AS400 | - | A | M |
| 171 | *M. acuminata* subsp*. siamea* | *siamea* | Mueang | Nan | SS & JS 402 | AS402 | - | A | M |
| 172 | *M. acuminata* subsp*. siamea* | *siamea* | Mueang | Prachinburi | SS & JS 411 | AS411 | - | A | M |
| 173 | *M. acuminata* subsp*. siamea* | *siamea* | Mae Taeng | Chiang Mai | SS & JS 415 | AS415 | L | A | M |
| 174 | *M. acuminata* subsp*. siamea* | *siamea* | Pai | Mae Hong Son | SS & JS 417 | AS417 | - | A | M |
| 175 | *M. acuminata* subsp*. siamea* | *siamea* | Sai Yok | Kanchanaburi | SS & JS 434 | AS434 | - | A | - |
| 176 | *M. acuminata* subsp*. siamea* | *siamea* | Suan Phueng | Ratchaburi | SS & JS 437 | AS437 | - | A | M |
| 177 | *M. acuminata* subsp*. siamea* | *siamea* | Suan Phueng | Ratchaburi | SS & JS 439 | AS439 | - | A | M |
| 178 | *M. acuminata* subsp*. siamea* | *siamea* | Si Sawat | Kanchanaburi | SS & JS 451 | AS451 | - | A | M |
| 179 | *M. acuminata* subsp*. siamea* | *siamea* | Sri Sawad | Kanchanaburi | SS & JS 453 | AS453 | L | A | M |
| 180 | *M. acuminata* subsp*. siamea* | *siamea* | Si Sawat | Kanchanaburi | SS & JS 454 | AS454 | - | A | M |
| 181 | *M. acuminata* subsp*. siamea* | *siamea* | Si Sawat | Kanchanaburi | SS & JS 455 | AS455 | - | A | M |
| 182 | *M. acuminata* subsp*. siamea* | *siamea* | Si Sawat | Kanchanaburi | SS & JS 456 | AS456 | - | A | M |
| 183 | *M. acuminata* subsp*. siamea* | *siamea* | Thong Pha Phum | Kanchanaburi | SS & JS 457 | AS457 | - | A | M |
| 184 | *M. acuminata* subsp*. siamea* | *siamea* | Thong Pha Phum | Kanchanaburi | SS & JS 458 | AS458 | L | A | M |
| 185 | *M. acuminata* subsp*. siamea* | *siamea* | Sai Yok | Kanchanaburi | SS & JS 463 | AS463 | - | A | - |
| 186 | *M. acuminata* subsp*. siamea* | *siamea* | Khao Kho | Phetchabun | SS & JS 465 | AS465 | L | A | M |
| 187 | *M. acuminata* subsp*. siamea* | *siamea* | Pad | Uttaradit | SS & JS 466 | AS466 | L | A | M |
| 188 | *M. acuminata* subsp*. siamea* | *siamea* | Santisuk | Nan | SS & JS 469 | AS469 | - | A | - |
| 189 | *M. acuminata* subsp*. siamea* | *siamea* | Santi Suk | Nan | SS & JS 470 | AS470 | L | A | M |
| 190 | *M. acuminata* subsp*. siamea* | *siamea* | Bo Kluea | Nan | SS & JS 474 | AS474 | - | A | M |
| 191 | *M. acuminata* subsp*. siamea* | *siamea* | Den Chai | Prae | SS & JS 476 | AS476 | L | A | M |
| 192 | *M. acuminata* subsp*. siamea* | *siamea* | Nakhon Thai | Phitsanulok | SS & JS 498 | AS498 | L | A | M |
| 193 | *M. acuminata* subsp*. siamea* | *siamea* | Wat Bot | Phitsanulok | SS & JS 499 | AS499 | L | A | M |
| 194 | *M. acuminata* subsp*. siamea* | *siamea* | Rong Kwang | Phrae | SS & JS 500 | AS500 | L | A | M |
| 195 | *M. acuminata* subsp*. siamea* | *siamea* | Mae Charim | Nan | SS & JS 506 | AS506 | - | A | - |
| 196 | *M. acuminata* subsp*. siamea* | *siamea* | Mueang | Phrae | SS & JS 510 | AS510 | - | A | - |
| 197 | *M. acuminata* subsp*. siamea* | *siamea* | Na Noi | Nan | SS & JS 529 | AS529 | - | A | - |
| 198 | *M. acuminata* subsp*. siamea* | *siamea* | Sai Yok | Kanchanaburi | SS & JS 537 | AS537 | - | A | M |
| 199 | *M. acuminata* subsp*. siamea* | *siamea* | Long | Phrae | SS & JS 549 | AS549 | - | A | M |
| 200 | *M. acuminata* subsp*. siamea* | *siamea* | Si Satchanalai | Sukhothai | SS & JS 557 | AS557 | - | A | M |
| 201 | *M. acuminata* subsp*. siamea* | *siamea* | Si Satchanalai | Sukhothai | SS & JS 558 | AS558 | - | A | M |
| 202 | *M. acuminata* subsp*. siamea* | *siamea* |  | Nan | SS & JS 669 | AS669 | - | A | - |
| 203 | *M. acuminata* subsp*. siamea* | *siamea* | Ban Na | Nakhon Nayok | SS & JS 702 | AS702 | - | A | - |
| 204 | *M. acuminata* subsp*. truncata* | *-* | Betong | Yala | SS & JS 206 | AT206 | L | - | M |
| 205 | *M. acuminata* subsp*. truncata* | *-* | Sukhirin | Narathiwat | SS & JS 490 | AT490 | L | - | M |
| 206 | *M. acuminata* subsp*. truncata* | *-* | Su-ngaipadi | Narathiwat | SS & JS 491 | AT491 | - | - | M |
| 207 | *M. acuminata* subsp*. truncata* | *-* | Waeng | Narathiwat | SS & JS 493 | AT493 | L | - | M |
| 208 | *M. acuminata* subsp*. truncata* | *-* | Si Sakhon | Narathiwat | SS & JS 495 | AT495 | - | - | M |
| 209 | *M. acuminata* subsp*. truncata* | *-* | Betong | Yala | SS & JS 497 | AT497 | L | - | M |
| 210 | *M. acuminata* subsp*. truncata* | *-* | Waeng | Narathiwat | SS & JS 698 | AT698 | L | - | M |
| 211 | *M. acuminata* subsp*.* *truncata* | *-* | Betong | Yala | SS & JS 700 | AT700 | L | - | M |
| 212 | *M. itinerans* | *-* | Doi Saket | Chiang Mai | SS & JS 259 | MI259 | - | - | M |
| 213 | *M. itinerans* | *-* | Umphang | Tak | SS & JS 313 | MI313 | - | - | M |
| 214 | *M. itinerans* | *-* | Sri Sawad | Kanchanaburi | SS & JS 452 | MI452 | - | - | M |
| 215 | *M. rubra* | *-* | Sai Yok | Kanchanaburi | SS & JS 240 | MR240 | - | - | M |
| 216 | *M. rubra* | *-* | Thong Pha Phum | Kanchanaburi | SS & JS 243 | MR243 | - | - | M |
| 217 | *M. rubra* | *-* | Mae Sot | Tak | SS & JS 308 | MR308 | - | - | M |
| 218 | *M. rubra* | *-* | Umphang | Tak | SS & JS 318 | MR318 | - | - | M |
| 219 | *M. rubra* | *-* | Khun Yuam | Mae Hong Son | SS & JS 352 | MR352 | - | - | M |
| 220 | *M. rubra* | *-* | Mae On | Chiang Mai | SS & JS 564 | MR564 | - | - | M |
| 221 | *M. rubra* | *-* | Chatuchak | Bangkok | SS & JS 566 | MR566 | - | - | - |
