# Supplementary material for: Delimitation of the wild banana Musa acuminata subspecific complex (Musaceae) in Thailand
Source: PhytoKeys. 2026 Aug 14;278:127–56. doi: 10.3897/phytokeys.278.165557 (PMC13498946; doi:10.3897/phytokeys.278.165557)
Supplement: Supplementary file 2 [file phytokeys-278-127_article-165557__-s002.docx]

**Table 2S** **(Supplement)** Data matrix of morphological characters used in multivariate analysis. Codings appeared based on those in Table 2. From all four subspecies found in Thailand, 195 Musa accessions were investigated.

Characters: 1 1 1 1 1 1 1

1 2 3 4 5 6 7 8 9 0 1 2 3 4 5 6

AK009	5 5 1 1 2 2 2 3 1 1 2 1 1 2 1 2

AK104	3 3 1 1 2 2 2 3 1 1 2 1 1 2 1 2

AK105	3 3 1 1 2 2 2 3 1 1 2 1 1 2 1 2

AK106	4 3 1 1 2 2 2 3 1 1 2 1 1 2 1 2

AK107	3 3 1 1 2 2 4 3 1 1 2 1 1 2 1 2

AK108	3 3 1 1 2 2 2 3 1 1 2 1 1 2 1 2

AK118	3 5 1 1 2 2 2 3 1 1 2 1 1 2 1 2

AK137	3 5 1 1 2 2 2 3 1 1 2 1 1 2 1 2

AK142	3 5 1 1 2 2 4 3 1 1 3 1 1 2 1 2

AK144	3 5 1 1 2 2 2 3 1 1 2 1 1 2 1 2

AK145	3 5 1 1 2 2 2 3 1 1 2 1 1 2 1 2

AK146	3 3 1 1 2 2 4 3 1 1 2 1 1 2 1 2

AK374	3 3 1 1 2 2 4 3 1 1 2 1 1 2 1 2

AK375	4 3 1 1 2 2 2 3 1 1 2 1 1 2 1 2

AK381	3 3 1 1 2 2 2 3 1 1 2 1 1 2 1 2

AK382	3 5 1 1 2 2 3 2 1 1 2 1 1 2 1 2

AK598	3 5 1 1 2 2 2 3 1 1 2 1 1 2 1 2

AK599	3 5 1 1 2 2 2 3 1 1 2 1 1 2 1 2

AK685	3 5 1 1 2 2 2 3 1 1 2 1 1 2 1 2

AK688	3 5 1 1 2 2 3 3 1 1 2 1 1 2 1 2

AK689	3 5 1 1 2 2 2 3 1 1 2 2 3 2 1 2

AK690	3 5 1 1 2 2 2 3 1 1 2 1 1 2 1 2

AK251	3 2 1 1 2 2 2 3 1 1 2 2 3 2 1 2

AK600	3 3 1 1 2 2 2 3 1 1 2 2 3 2 1 2

Characters: 1 1 1 1 1 1 1

1 2 3 4 5 6 7 8 9 0 1 2 3 4 5 6

AK601	3 5 1 1 2 2 2 3 1 1 2 2 3 2 1 2

AK602	3 5 1 1 2 2 2 3 1 1 2 1 1 2 1 2

AM005	5 5 1 1 2 1 3 2 1 2 1 1 1 2 1 2

AM006	5 5 1 1 2 1 4 2 1 2 1 1 1 2 1 2

AM010	3 5 1 1 2 1 3 2 1 2 1 1 1 2 1 2

AM109	4 5 1 1 2 1 3 2 1 2 1 1 1 2 1 2

AM111	3 5 1 1 2 1 3 2 1 2 1 1 1 2 1 2

AM112	3 5 1 1 2 1 2 2 1 1 1 1 1 2 1 2

AM113	3 5 1 1 2 1 3 2 1 2 1 1 1 2 1 2

AM114	3 3 1 1 2 1 3 2 1 2 1 1 1 2 1 2

AM115	3 5 1 1 2 1 3 2 1 2 1 1 1 2 1 2

AM116	3 3 1 1 2 1 1 1 1 2 1 1 1 2 1 2

AM138	3 5 1 1 2 1 4 2 1 1 1 1 1 2 1 2

AM139	3 5 1 1 2 1 3 2 1 2 1 1 1 2 1 2

AM140	3 3 1 1 2 1 3 2 1 2 1 1 1 2 1 2

AM141	3 3 1 1 2 1 3 2 1 2 1 1 1 2 1 2

AM199	3 5 1 1 2 1 3 2 1 2 1 1 1 2 1 2

AM200	3 5 1 1 2 1 3 2 1 1 1 1 1 2 1 2

AM202	3 5 1 1 2 1 3 1 1 1 1 1 1 2 1 2

AM203	3 5 1 1 2 1 3 2 1 1 1 1 1 2 1 2

AM204	3 5 1 1 2 1 3 2 1 1 1 1 1 2 1 2

AM205	3 5 1 1 2 1 3 2 1 1 1 1 1 2 1 2

AM209	3 5 1 1 2 1 1 1 1 1 1 1 1 2 1 2

AM210	3 5 1 1 2 1 3 2 1 2 1 1 1 2 1 2

AM211	3 5 1 1 2 1 3 2 1 1 2 1 1 2 1 2

AM212	3 5 1 1 2 1 3 1 1 1 1 1 1 2 1 2

AM213	3 5 1 1 2 1 3 2 1 1 2 1 1 2 1 2

Characters: 1 1 1 1 1 1 1

1 2 3 4 5 6 7 8 9 0 1 2 3 4 5 6

AM214	3 5 1 1 2 1 3 2 1 1 1 1 1 2 1 2

AM216	3 5 1 1 2 1 3 2 1 1 1 1 1 2 1 2

AM217	3 5 1 1 2 1 3 2 1 1 1 1 1 2 1 2

AM218	3 5 1 1 2 1 3 2 1 1 1 1 1 2 1 2

AM219	3 5 1 1 2 1 3 1 1 2 1 1 1 2 1 2

AM220	3 3 1 1 2 1 3 2 1 1 1 1 1 2 1 2

AM228	3 5 1 1 2 1 3 2 1 2 1 1 1 2 1 2

AM274	3 5 1 1 2 1 3 2 1 2 1 1 1 2 1 2

AM281	5 2 1 1 2 1 3 2 1 2 1 1 1 2 1 2

AM309	3 2 1 1 2 1 3 2 1 1 1 1 1 2 1 2

AM357	3 2 1 1 2 1 3 1 1 1 1 1 1 2 1 2

AM376	4 5 1 1 2 1 1 1 1 1 2 1 1 2 1 2

AM377	3 3 1 1 2 1 1 1 1 1 1 1 1 2 1 2

AM378	3 2 1 1 2 1 4 2 1 1 1 1 1 2 1 2

AM379	3 2 1 1 2 1 1 1 1 1 1 1 1 2 1 2

AM380	3 5 1 1 2 1 4 2 1 2 1 1 1 2 1 2

AM445	4 2 1 1 2 1 1 1 1 1 1 1 1 2 1 2

AM477	3 5 1 1 2 1 3 1 1 1 2 1 1 2 1 2

AM478	3 2 1 1 2 1 3 2 1 2 1 1 1 2 1 2

AM482	3 5 1 1 2 1 1 1 1 1 1 1 1 2 1 2

AM485	3 5 1 1 2 1 1 1 1 1 2 1 1 2 1 2

AM489	3 3 1 1 2 1 1 1 1 1 1 1 1 2 1 2

AM496	3 3 1 1 2 1 2 2 1 1 2 1 1 2 1 2

AM546	3 4 1 1 2 1 1 1 1 1 1 1 1 2 1 2

AM691	3 5 1 1 2 1 3 2 1 2 1 1 1 2 1 2

AM692	3 5 1 1 2 1 3 2 1 2 1 1 1 2 1 2

AM699	3 5 1 1 2 1 3 2 1 1 1 1 1 2 1 2

Characters: 1 1 1 1 1 1 1

1 2 3 4 5 6 7 8 9 0 1 2 3 4 5 6

AS173	3 2 1 1 2 1 1 1 1 1 3 2 3 2 1 2

AS177	3 2 1 1 2 1 1 1 1 1 3 2 3 2 1 2

AS224	3 2 1 1 2 1 3 2 1 1 3 2 3 2 1 2

AS307	1 2 1 1 2 1 1 1 1 2 3 2 3 2 1 2

AS338	3 2 1 1 2 ? 1 1 2 1 3 1 1 2 ? ?

AS442	3 2 1 1 2 ? 1 1 2 1 3 1 1 2 ? ?

AS001	1 2 1 1 2 1 1 1 1 1 3 1 1 2 1 2

AS007	3 2 1 1 2 1 1 1 1 1 3 1 1 2 1 2

AS008	3 2 1 1 2 1 1 1 1 1 3 1 1 2 1 2

AS019	3 2 1 1 2 1 3 1 1 1 3 1 1 2 1 2

AS021	1 2 1 1 2 1 1 1 1 1 3 1 1 2 1 2

AS089	1 2 1 1 2 1 1 1 1 1 3 1 1 2 1 2

AS090	3 2 1 1 2 1 1 1 1 1 3 1 1 2 2 2

AS091	1 2 1 1 2 1 1 1 1 1 3 1 1 2 1 2

AS092	3 2 1 1 2 1 1 1 1 1 3 1 1 2 1 2

AS094	3 2 1 1 2 1 1 1 1 1 3 1 1 2 1 2

AS096	3 2 1 1 2 1 1 1 1 1 3 1 1 2 1 2

AS097	2 2 1 1 2 1 1 1 1 1 3 1 1 2 1 2

AS098	2 2 1 1 2 1 1 1 1 1 3 1 1 2 1 2

AS099	3 2 1 1 2 1 1 1 1 1 3 1 1 2 1 2

AS101	2 2 1 1 2 1 1 1 1 1 3 1 1 2 1 2

AS102	3 2 1 1 2 1 1 1 1 1 3 1 1 2 1 2

AS126	3 2 1 1 2 1 1 1 1 1 3 1 1 2 1 2

AS129	1 2 1 1 2 1 1 1 1 1 3 1 1 2 1 2

AS131	3 2 1 1 2 1 1 1 1 1 3 1 1 2 1 2

AS132	3 2 1 1 2 1 1 1 1 1 3 1 1 2 1 2

AS136	3 2 1 1 2 1 1 1 1 1 3 1 1 2 1 2

Characters: 1 1 1 1 1 1 1

1 2 3 4 5 6 7 8 9 0 1 2 3 4 5 6

AS147	3 2 1 1 2 1 1 1 1 1 3 1 1 2 1 2

AS172	2 2 1 1 2 1 1 1 1 1 3 1 1 2 1 2

AS174	3 2 1 1 2 1 3 1 1 1 3 1 1 2 1 2

AS180	3 2 1 1 2 1 1 1 1 1 3 1 1 2 1 2

AS181	3 2 1 1 2 1 1 1 1 1 3 1 1 2 1 2

AS182	2 2 1 1 2 1 1 1 1 1 3 1 1 2 1 2

AS185	1 2 1 1 2 1 1 1 1 1 3 1 1 2 1 2

AS197	3 2 1 1 2 1 1 1 1 1 3 1 1 2 1 2

AS221	1 2 1 1 2 1 1 1 1 1 3 1 1 2 1 2

AS225	3 2 1 1 2 1 1 1 1 1 3 1 1 2 1 2

AS227	2 2 1 1 2 1 1 1 1 1 3 1 1 2 1 2

AS232	3 2 1 1 2 1 1 1 1 1 3 1 1 2 1 2

AS233	2 2 1 1 2 1 1 1 1 1 3 1 1 2 1 2

AS238	3 2 1 1 2 1 1 1 1 1 3 1 1 2 1 2

AS245	3 2 1 1 2 1 1 1 1 1 3 1 1 2 1 2

AS247	3 2 1 1 2 1 3 2 1 1 3 1 1 2 1 2

AS250	2 2 1 1 2 1 1 1 1 1 3 1 1 2 1 2

AS258	1 2 1 1 2 1 1 1 1 1 3 1 1 2 1 2

AS262	3 1 1 1 2 1 1 1 1 1 3 1 1 2 1 2

AS264	2 1 1 1 2 1 1 1 1 1 3 1 1 2 1 2

AS266	3 2 1 1 2 1 1 1 1 1 3 1 1 2 1 2

AS268	3 2 1 1 2 1 1 1 1 1 3 1 1 2 1 2

AS283	3 2 1 1 2 1 1 1 1 1 3 1 1 2 1 2

AS286	2 2 1 1 2 1 1 1 1 1 3 1 1 2 1 2

AS289	1 2 1 1 2 1 1 1 1 1 3 1 1 2 1 2

AS290	4 2 1 1 2 1 1 1 1 1 3 1 1 2 1 2

AS291	3 2 1 1 2 1 1 1 1 1 3 1 1 2 1 2

Characters: 1 1 1 1 1 1 1

1 2 3 4 5 6 7 8 9 0 1 2 3 4 5 6

AS293	3 2 1 1 2 1 1 1 1 1 3 1 1 2 1 2

AS300	3 2 1 1 2 1 1 1 1 1 3 1 1 2 1 2

AS301	3 2 1 1 2 1 1 1 1 1 3 1 1 2 1 2

AS303	3 2 1 1 2 1 1 1 1 1 3 1 1 2 1 2

AS305	3 2 1 1 2 1 1 1 1 1 3 1 1 2 1 2

AS310	2 2 1 1 2 1 1 1 1 1 3 1 1 2 1 2

AS312	3 2 1 1 2 1 1 1 1 1 3 1 1 2 1 2

AS320	1 2 1 1 2 1 1 1 1 1 3 1 1 2 1 2

AS321	4 2 1 1 2 1 1 1 1 1 3 1 1 2 1 2

AS322	3 2 1 1 2 1 1 1 1 1 3 1 1 2 1 2

AS325	3 2 1 1 2 1 3 1 1 1 3 1 1 2 1 2

AS326	3 2 1 1 2 1 1 1 1 1 3 1 1 2 1 2

AS330	3 1 1 1 2 1 1 1 1 1 3 1 1 2 1 2

AS334	3 1 1 1 2 1 1 1 1 1 3 1 1 2 1 2

AS339	3 2 1 1 2 1 1 1 1 1 3 1 1 2 1 2

AS343	3 2 1 1 2 1 1 1 1 1 3 1 1 2 1 2

AS372	3 2 1 1 2 1 1 1 1 1 3 1 1 2 1 2

AS373	3 2 1 1 2 1 1 1 1 1 3 1 1 2 1 2

AS390	3 1 1 1 2 1 1 1 1 1 3 1 1 2 1 2

AS392	3 2 1 1 2 1 1 1 1 1 3 1 1 2 1 2

AS394	1 2 1 1 2 1 1 1 1 1 3 1 1 2 1 2

AS400	2 2 1 1 2 1 1 1 1 1 3 1 1 2 1 2

AS402	2 2 1 1 2 1 1 1 1 1 3 1 1 2 1 2

AS411	2 2 1 1 2 1 1 1 1 1 3 1 1 2 1 2

AS415	2 2 1 1 2 1 1 1 1 1 3 1 1 2 1 2

AS417	1 1 1 1 2 1 1 1 1 1 3 1 1 2 1 2

AS437	3 2 1 1 2 1 1 1 1 1 3 1 1 2 1 2

Characters: 1 1 1 1 1 1 1

1 2 3 4 5 6 7 8 9 0 1 2 3 4 5 6

AS439	3 2 1 1 2 1 1 1 1 1 3 1 1 2 1 2

AS451	3 2 1 1 2 1 1 1 1 1 3 1 1 2 1 2

AS453	3 2 1 1 2 1 1 1 1 1 3 1 1 2 1 2

AS454	1 2 1 1 2 1 1 1 1 1 3 1 1 2 1 2

AS455	3 2 1 1 2 1 1 1 1 1 3 1 1 2 1 2

AS456	2 2 1 1 2 1 1 1 1 1 3 1 1 2 1 2

AS457	2 2 1 1 2 1 1 1 1 1 3 1 1 2 1 2

AS458	2 2 1 1 2 1 1 1 1 1 3 1 1 2 1 2

AS465	2 2 3 1 2 1 1 1 1 1 3 1 1 2 1 2

AS466	3 2 3 1 2 1 1 1 1 1 3 1 1 2 1 2

AS470	3 2 1 1 2 1 1 1 1 1 3 1 1 2 1 2

AS474	3 2 1 1 2 1 1 1 1 1 3 1 1 2 1 2

AS476	3 2 1 1 2 1 1 1 1 1 3 1 1 2 1 2

AS498	2 2 1 1 2 1 1 1 1 1 3 1 1 2 1 2

AS499	2 2 1 1 2 1 1 1 1 1 3 1 1 2 1 2

AS500	2 2 1 1 2 1 1 1 1 1 3 1 1 2 1 2

AS537	2 2 1 1 2 1 1 1 1 1 3 1 1 2 1 2

AS549	2 2 1 1 2 1 1 1 1 1 3 1 1 2 1 2

AS557	3 1 1 1 2 1 1 1 1 1 3 1 1 2 1 2

AS558	3 1 1 1 2 1 1 1 1 1 3 1 1 2 1 2

AT206	3 5 2 1 2 1 1 1 1 1 1 3 1 2 1 2

AT490	3 5 2 1 2 1 1 1 1 1 1 3 1 2 1 2

AT491	4 5 2 1 2 1 1 1 1 1 1 3 1 2 1 2

AT493	3 5 2 1 2 1 1 1 1 1 1 3 1 2 1 2

AT495	3 5 2 1 2 1 1 1 1 1 1 3 1 2 1 2

AT497	3 5 2 1 2 1 1 1 1 1 1 3 1 2 1 2

AT698	3 5 2 1 2 1 1 1 1 1 1 3 1 2 1 2

Characters: 1 1 1 1 1 1 1

1 2 3 4 5 6 7 8 9 0 1 2 3 4 5 6

MI259	3 5 1 1 2 1 1 1 1 1 1 3 1 1 2 2

MI313	5 5 1 1 2 1 1 1 1 1 1 3 1 1 2 2

MI452	3 5 1 1 2 1 1 1 1 1 1 3 1 1 2 2

MR240	2 1 1 2 1 1 5 4 1 1 2 4 2 2 1 1

MR243	1 2 1 2 1 1 5 4 1 1 2 4 2 2 1 1

MR308	1 1 1 2 1 1 5 4 1 1 2 4 2 2 1 1

MR318	1 1 1 2 1 1 5 4 1 1 1 4 2 2 1 1

MR352	1 2 1 2 1 1 5 4 1 1 2 4 2 2 1 1

MR564	1 1 1 2 1 1 5 4 1 1 2 4 2 2 1 2
